# Supplementary material for: Non-Abelian three-loop braiding statistics for 3D fermionic topological phases
Source: Nat Commun. 2021 May 27;12:3191. doi: 10.1038/s41467-021-23309-3 (PMC8159978; doi:10.1038/s41467-021-23309-3)
Supplement: Supplementary file 1 — Supplementary Information [file 41467_2021_23309_MOESM1_ESM.pdf]

Non-Abelian Three-Loop Braiding Statistics for 3D Fermionic Topological Phases

Jing-Ren Zhou,[1,*] Qing-Rui Wang,[1,*] Chenjie Wang,[2,†] and Zheng-Cheng Gu[1,‡]

[1]*Department of Physics, The Chinese University of Hong Kong, Shatin, New Territories, Hong Kong*
[2]*Department of Physics and HKU-UCAS Joint Institute for Theoretical and Computational Physics, The University of Hong Kong, Pokfulam Road, Hong Kong, China*

I. SUPPLEMENTARY NOTE 1: SOME BASIC PROPERTIES OF THE FUSION RULE

There are several properties for the generally nontrivial fusion rule $\alpha \times \beta = \sum_{\delta} N_{\alpha\beta}^{\delta}\delta$ in our gauged FSPT system, where α, β, δ are all loop-like excitations, and the proofs are the same as the bosonic case given in Ref[1]. The properties are:

(1) For any fusion channel δ:

$$\phi_\delta = \phi_\alpha + \phi_\beta \tag{1}$$

which means that different fusion channels only differ by their attached charges.

(2) When a loop α is fused with a charge q, there is exactly one fusion outcome:

$$q \times \alpha = \alpha' \tag{2}$$

(3) The fusion multiplicity $N_{\alpha\overline{\alpha}}^{q} = 0, 1$, where q is any charge in the fusion channels of α and $\overline{\alpha}$.

(4) If $\phi_{\alpha'} = \phi_\alpha$ and $\phi_{\beta'} = \phi_\beta$, then there exist charges q_1 and q_2 such that $\alpha' = \alpha \times q_1$, $\beta' = \beta \times q_2$ and $\delta' = \delta \times q_1 \times q_2$.

II. SUPPLEMENTARY NOTE 2: SOME BASIC DEFINITIONS

Definition: The *fusion space* $V_{\alpha\beta,c}^{\delta}$ that fuses two loops α, β into a single fusion channel δ with base loop c, is a Hilbert space spanned by the set of orthogonal basis[2,3]:

$$\{|\alpha\beta, c; \delta, \mu\rangle \,|\mu = 1, ..., N_{\alpha\beta,c}^{\delta}\} \tag{3}$$

which can be simplified as $\{|\alpha\beta, c; \delta\rangle\}$ as $N_{\alpha\beta,c}^{\delta}$ is always 1 in our theory. And the full Hilbert space for the fusion of α, β with base loop c is:

$$V_{\alpha\beta,c} \cong \bigoplus_{\delta} V_{\alpha\beta,c}^{\delta} \tag{4}$$

Accordingly the *spliting space* for a single fusion channel δ is spanned by the dual basis:

$$\{\langle\alpha\beta, c; \delta|\} \tag{5}$$

Definition: Consider a local system involving only two loops α, β both linked to a base loop γ, and their fusion outcome δ is known. The *Abelian R-symbol* $R_{\alpha\beta,c}^{\delta}$ that exchanges two loops α, β, during which their fusion channel δ is fixed, is defined as a map[2,3]:

$$R_{\alpha\beta,c}^{\delta} : V_{\alpha\beta,c}^{\delta} \to V_{\beta\alpha,c}^{\delta} \tag{6}$$

$$|\beta\alpha, c; \delta\rangle = R_{\alpha\beta,c}^{\delta} \,|\alpha\beta, c; \delta\rangle \tag{7}$$

which is a basis-dependent pure phase, as $|\alpha\beta, c; \delta\rangle$ may differ $|\beta\alpha, c; \delta\rangle$ by a gauge transformation. Specially, the R-symbol $R_{\alpha\alpha,c}^{\delta} : V_{\alpha\alpha,c}^{\delta} \to V_{\alpha\alpha,c}^{\delta}$ exchanging two identical loops is basis-independent.

Definition: The *non-Abelian R-symbol* $R_{\alpha\beta,c}$ is defined as a matrix:

$$R_{\alpha\beta,c} : \bigoplus_{\delta} V_{\alpha\beta,c}^{\delta} \to \bigoplus_{\delta} V_{\beta\alpha,c}^{\delta} \tag{8}$$

which can be diagonalized by choosing a proper basis if there is no other fusion process involved:

$$R_{\alpha\beta,c} = \begin{bmatrix} R_{\alpha\beta,c}^{\delta_1} & 0 & \\ 0 & R_{\alpha\beta,c}^{\delta_2} & \\ & & \ddots \end{bmatrix} \tag{9}$$

where $\delta_1, \delta_2, ...$ are all the possible fusion channels of α and β.

Example: For 2D Ising anyons[2], which contain anyon types $\{1, \sigma, \psi\}$,

$$R_{\sigma\sigma} = \mathcal{X} e^{-i\nu\pi/8} \begin{bmatrix} 1 & 0 \\ 0 & i \end{bmatrix} \tag{10}$$

where $\mathcal{X}$ is the Frobenius-Schur indicator, and the Chern number ν is odd (mod 16) for non-Abelian Ising anyons.

Definition: For the same system above, similarly the *Abelian B-symbol* $B_{\alpha\beta,c}^{\delta}$ that braids loop α around β linked to a base loop γ is defined as:

$$B_{\alpha\beta,c}^{\delta} = R_{\beta\alpha,c}^{\delta} R_{\alpha\beta,c}^{\delta} : V_{\alpha\beta,c}^{\delta} \to V_{\alpha\beta,c}^{\delta} \tag{11}$$

which is basis-independent as it maps between the same fusion space.

Definition: The *non-Abelian B-symbol* $B_{\alpha\beta,c}$ is defined as:

$$B_{\alpha\beta,c} : \bigoplus_{\delta} V_{\alpha\beta,c}^{\delta} \to \bigoplus_{\delta} V_{\alpha\beta,c}^{\delta} \tag{12}$$

which can be diagonalized by choosing a proper basis if there is no other fusion process involved:

$$B_{\alpha\beta,c} = \begin{bmatrix} B_{\alpha\beta,c}^{\delta_1} & 0 & \\ 0 & B_{\alpha\beta,c}^{\delta_2} & \\ & & \ddots \end{bmatrix} \tag{13}$$

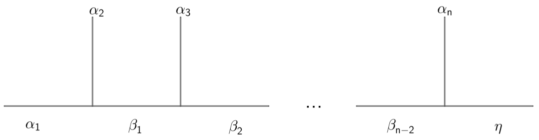

Supplementary Figure. 1. **The diagrammatic expression of the standard basis.** We choose a specific fusion order that firstly fusing α_1 and α_2, then fusing the result β_1 with α_3, then fusing the result β_2 with α_4, and so on.

Example: For 2D Ising anyons,

$$B_{\sigma\sigma} = e^{-i\nu\pi/4} \begin{bmatrix} 1 & 0 \\ 0 & -1 \end{bmatrix} \tag{14}$$

Definition: Consider a local system involving three loops α, β, ϵ all linked to a base loop γ, whose total fusion outcome η is known. The *F-symbol* $F^{\eta}_{\epsilon\alpha\beta,c}$ that maps between two different fusion ways, is defined as a generally non-diagonalized matrix[2,3]:

$$F^{\eta}_{\epsilon\alpha\beta,c} : \bigoplus_{\delta}(V^{\delta}_{\alpha\beta,c} \otimes V^{\eta}_{\delta\epsilon,c}) \rightarrow \bigoplus_{\rho}(V^{\rho}_{\epsilon\alpha,c} \otimes V^{\eta}_{\rho\beta,c}) \tag{15}$$

$$|\rho\beta, c; \eta\rangle \, |\alpha\epsilon, c; \rho\rangle = \sum_{\delta} (F^{\eta}_{\epsilon\alpha\beta,c})^{\rho}_{\delta} \, |\delta\epsilon, c; \eta\rangle \, |\alpha\beta, c; \delta\rangle \tag{16}$$

Example: For 2D Ising anyons,

$$F^{\sigma}_{\sigma\sigma\sigma} = \frac{\mathcal{X}}{\sqrt{2}} \begin{bmatrix} 1 & 1 \\ 1 & -1 \end{bmatrix} \tag{17}$$

Definition: Consider n loops $\alpha_1, \alpha_2, ..., \alpha_n$ all linked to a base loop γ, where the total fusion outcome η of the n loops is known. Then we define a *standard basis* in the total fusion space by specifying a particular fusion order[3]. For example, firstly fusing α_1 and α_2, then fusing the result with α_3, then fusing the result with α_4, and so on. The total fusion space can therefore be decomposited as:

$$V^{\eta}_{\alpha_1,...,\alpha_n,c} \cong \bigoplus_{\beta_1,...,\beta_{n-2}} V^{\beta_1}_{\alpha_1\alpha_2,c} \otimes V^{\beta_2}_{\beta_1\alpha_3,c} \otimes ... \otimes V^{\eta}_{\beta_{n-2}\alpha_n,c} \tag{18}$$

which is equivalently expressed by the diagram in Supplementary Figure.1.

Definition: Consider a local system involving three loops α, β, ϵ all linked to a base loop γ, where the total fusion outcome of the three loops η is known. The *$\widetilde{R}$-matrix* $\widetilde{R}^{\eta}_{\epsilon\alpha\beta,c}$ that exchanges two loops α, β, while it is diagonalized in the fusion space of ϵ and α, is defined as a generally non-diagonalized matrix[4]:

$$\widetilde{R}^{\eta}_{\epsilon\alpha\beta,c} : \bigoplus_{\delta}(V^{\delta}_{\epsilon\beta,c} \otimes V^{\eta}_{\delta\alpha,c}) \rightarrow \bigoplus_{\rho}(V^{\rho}_{\epsilon\beta,c} \otimes V^{\eta}_{\rho\alpha,c}) \tag{19}$$

$$\widetilde{R}^{\eta}_{\epsilon\alpha\beta,c} = F^{\eta}_{\epsilon\beta\alpha,c} R_{\alpha\beta,c} (F^{\eta}_{\epsilon\alpha\beta,c})^{-1} \tag{20}$$

Definition: For the same system above, similarly the *$\widetilde{B}$-matrix* $\widetilde{B}^{\eta}_{\epsilon\alpha\beta,c}$ that braids loop α around β, while it is diagonalized in the fusion space of ϵ and α, is defined as a generally non-diagonalized matrix:

$$\widetilde{B}^{\eta}_{\epsilon\alpha\beta,c} : \bigoplus_{\delta}(V^{\delta}_{\epsilon\alpha,c} \otimes V^{\eta}_{\delta\beta,c}) \rightarrow \bigoplus_{\rho}(V^{\rho}_{\epsilon\alpha,c} \otimes V^{\eta}_{\rho\beta,c}) \tag{21}$$

$$\widetilde{B}^{\eta}_{\epsilon\alpha\beta,c} = \widetilde{R}_{\beta\alpha,c}\widetilde{R}_{\alpha\beta,c} = F^{\eta}_{\epsilon\alpha\beta,c} R_{\beta\alpha,c} (F^{\eta}_{\epsilon\beta\alpha,c})^{-1} F^{\eta}_{\epsilon\beta\alpha,c} R_{\alpha\beta,c} (F^{\eta}_{\epsilon\alpha\beta,c})^{-1} = F^{\eta}_{\epsilon\alpha\beta,c} B_{\alpha\beta,c} (F^{\eta}_{\epsilon\alpha\beta,c})^{-1} \tag{22}$$

Example: For 2D Ising anyons,

$$\widetilde{B}^{\sigma}_{\sigma\sigma\sigma} : (V^{1}_{\sigma\sigma} \otimes V^{\sigma}_{1\sigma}) \oplus (V^{\psi}_{\sigma\sigma} \otimes V^{\sigma}_{\psi\sigma}) \rightarrow (V^{1}_{\sigma\sigma} \otimes V^{\sigma}_{1\sigma}) \oplus (V^{\psi}_{\sigma\sigma} \otimes V^{\sigma}_{\psi\sigma}) \tag{23}$$

$$\widetilde{B}^{\sigma}_{\sigma\sigma\sigma} = e^{-i\nu\pi/4} \begin{bmatrix} 0 & 1 \\ 1 & 0 \end{bmatrix} \tag{24}$$

Definition: The *fusion matrix* for a loop α linked to a base loop γ is defined as[5,6]:

$$\widehat{N}_{\alpha,c} = (N^{\delta}_{\alpha\beta,c} : \beta, \delta \in M) \tag{25}$$

where M is a finite set called *superselection sectors*, which is the set of all distinguishable particle types in a theory.

Definition: The *quantum dimension* of a loop α linked to a base loop γ is defined as the largest eigenvalue of the fusion matrix $\widehat{N}_{\alpha,c}$, which can be understood thorugh a key property[2]:

$$d_{\alpha,c} d_{\beta,c} = \sum_{\delta} N^{\delta}_{\alpha\beta,c} d_{\delta,c}$$

which implies that the fusion matrix $\widehat{N}_{\alpha,c}$ has an eigenvector $v = (d_{\delta,c} : \delta \in M)$ and the corresponding eigenvalue is $d_{\alpha,c}$. According to Perron-Frobenius theorem, $d_{\alpha,c}$ is the largest eigenvalue of $\widehat{N}_{\alpha,c}$. Intuitively, quantum dimension is the intrinsic degree of freedom carried by an anyon.

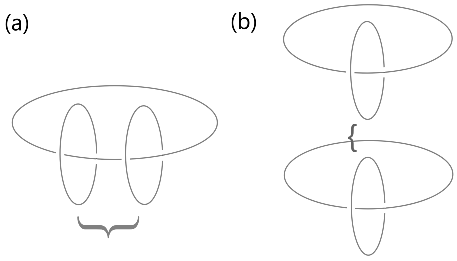

Supplementary Figure. 2. (a) **The "horizontal" fusion.** We fuse two "horizontal" loops that are both linked to a base loop. (b) **The "vertical fusion".** We fuse two "vertical" loops. After the "vertical" fusion, the two base loops are put together.

III. SUPPLEMENTARY NOTE 3: PROOF OF THE CONSTRAINTS

A. 3D "Vertical" Fusion Rule

In order to prove some of the newly involved 3D constraints, we need to consider a new kind of "vertical" fusion in analogy to the original "horizontal fusion", as shown in Supplementary Figure.2 (a) and (b). Consider two Hopf-link systems, where the two loops in dfferent systems are in the same type. Then the *3D "vertical" fusion rule* has the form:

$$\xi^1_{\mu,\sigma_1} \circ \xi^2_{\mu,\sigma_2} = \xi'_{\mu,(\sigma_1+\sigma_2)} + \xi''_{\mu,(\sigma_1+\sigma_2)} + ... \tag{26}$$

where the "vertical" fusion is denoted as "$\circ$". The fusion outcomes have the same flux but different attached charges Q, and the $+$ in $(\sigma_1+\sigma_2)$ means only putting two loops together, which applies when fusing the loops or not does not matter as the charges attached on a base loop do not affect the three-loop braiding process. And this "vertical" fusion rule can be understood in a way that the two loops that are about to fuse annihilate at a point (as particle and antiparticle) to vacuum or some charge Q. And if the fusion outcome is a charge Q, it will be attached to the loop after fusion.

First we would like to mention that the expression of the topological invariant $\Theta_{\mu\nu,\sigma}$ can be further written as:

$$e^{i\Theta_{\mu\nu,\sigma}} I = (B_{\xi_\mu\xi_\nu,e_\sigma})^{N^{\mu\nu}} = (B^{\delta}_{\xi_\mu\xi_\nu,e_\sigma})^{N^{\mu\nu}} I \tag{27}$$

where the fusion channel δ is arbitrary, as the result is the same for all fusion channels[7], and I is the identity matrix in the fusion space $\bigoplus_{\delta} V^{\delta}_{\xi_\mu\xi_\nu,c}$.

Then we consider two three-loop systems, where the three loops in different systems are all in the same type as shown in Supplementary Figure.3. Specifically, before the "vertical" fusions, we choose to fix the fusion channel for each three-loop system. Thereby the braiding operator for the whole system before "vertical" fusions are:

$$B^{\delta_1}_{\xi^1_\mu\xi^1_\nu,e^1_\sigma} B^{\delta_2}_{\xi^2_\mu\xi^2_\nu,e^2_\sigma} \tag{28}$$

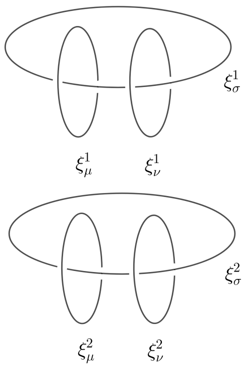

Supplementary Figure. 3. **The "vertical" fusion of two three-loop systems.** We "vertically" fuse loop ξ^1_μ with ξ^2_μ, loop ξ^1_ν with ξ^2_ν. And the two base loops ξ^1_σ and ξ^2_σ are put together to be the base loop after fusion.

While after the "vertical" fusions, the braiding operator is:

$$\begin{bmatrix} B^{\delta'}_{\xi'_\mu\xi'_\nu,(e^1_\sigma+e^2_\sigma)} & 0 & \\ 0 & B^{\delta''}_{\xi''_\mu\xi''_\nu,(e^1_\sigma+e^2_\sigma)} & \\ & & \ddots \end{bmatrix} \tag{29}$$

where the vertical fusions $\xi^1_{\mu,\sigma_1} \circ \xi^2_{\mu,\sigma_2} = \xi'_{\mu,(\sigma_1+\sigma_2)} + \xi''_{\mu,(\sigma_1+\sigma_2)} + ...$ and $\xi^1_{\nu,\sigma_1} \circ \xi^2_{\nu,\sigma_2} = \xi'_{\nu,(\sigma_1+\sigma_2)} + \xi''_{\nu,(\sigma_1+\sigma_2)} + ...$ both generally have multiple fusion outcomes. According to the 4th property in section I, the fusion outcomes of the two loops after "vertical" fusions $\xi_{\mu,(\sigma_1+\sigma_2)}$ and $\xi_{\nu,(\sigma_1+\sigma_2)}$ are also multiple. And we can choose a particular basis in the fusion space such that the braiding operator is diagonalized.

Then we do the braiding processes for both cases (before and after "vertical" fusions) for $N^{\mu\nu}$ times, we obtain an equation:

$$(B^{\delta_1}_{\xi^1_\mu\xi^1_\nu,e^1_\sigma})^{N^{\mu\nu}} (B^{\delta_2}_{\xi^2_\mu\xi^2_\nu,e^2_\sigma})^{N^{\mu\nu}} = (B^{\delta'}_{\xi'_\mu\xi'_\nu,(e^1_\sigma+e^2_\sigma)})^{N^{\mu\nu}} \tag{30}$$

where $B^{\delta'}_{\xi'_\mu\xi'_\nu,(e^1_\sigma+e^2_\sigma)}$ is any of the diagonalized entry in the matrix (29). The eqn. (30) is equivalent to the claim that:

The $N^{\mu\nu}$ times of braiding as a whole commutes with the "vertical" fusions.

The proof of eqn.(30) is given as the following: Firstly the $N^{\mu\nu}$ times of braiding can be equivalently viewed as a successive braiding of $N^{\mu\nu}$ identical loops. As the $N^{\mu\nu}$ times of braiding eliminates the difference between different fusion channels, the $N^{\mu\nu}$ loops as a whole is actually an Abelian object, as shown in Supplementary Figure.4. And the remaining proof is similar as the Fig.6 in Ref[8].

Notice that the whole argument does not violate the conservation of anyon charge, as we have only specified the fusion channels but not the total charge of the initial state. And the exchanging operator for a loop with

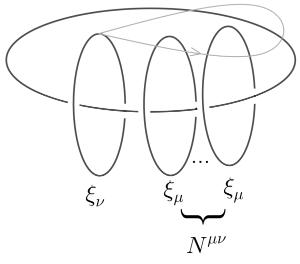

Supplementary Figure. 4. **The $N^{\mu\nu}$ loops as a whole can be viewed as an Abelian object.** This figure shows a braiding process of loop ξ_ν around $N^{\mu\nu}$ copies of ξ_μ loops, which commutes with the "vertical" fusions.

its anti-loop, i.e. the R-operator in the vacuum fusion channel, has a similar property if we do the exchanging processes for $\tilde{N}_\mu$ times:

$$(R^0_{\xi^1_\mu\bar{\xi}^1_\mu,e^1_\sigma})^{\tilde{N}_\mu}(R^0_{\xi^2_\mu\bar{\xi}^2_\mu,e^2_\sigma})^{\tilde{N}_\mu} = (R^0_{\xi'_\mu\bar{\xi}'_\mu,(e^1_\sigma+e^2_\sigma)})^{\tilde{N}_\mu} \tag{31}$$

where although the fusion channels of the two ξ^1_μ loops or two ξ^2_μ loops are both 0, the total fusion outcome of the four loops may not be 0, i.e. generally the right-hand side of (31) should be $(R^{\delta'}_{\xi'_\mu\bar{\xi}'_\mu,(e^1_\sigma+e^2_\sigma)})^{\tilde{N}_\mu}$. But as $(R^{\delta'}_{\xi'_\mu\bar{\xi}'_\mu,(e^1_\sigma+e^2_\sigma)})^{\tilde{N}_\mu} = (R^0_{\xi'_\mu\bar{\xi}'_\mu,(e^1_\sigma+e^2_\sigma)})^{\tilde{N}_\mu}$ due to the $\tilde{N}_\mu$ times of exchanging, we can write $(R^0_{\xi'_\mu\bar{\xi}'_\mu,(e^1_\sigma+e^2_\sigma)})^{\tilde{N}_\mu}$ at the right-hand side of (31) safely.

B. Linear Properties of the Topological Invariants

The linear properties of the braiding processes that are useful in proving the newly involved 3D constraints are:

$$\Theta_{(\mu_1\circ\mu_2),(\sigma_1+\sigma_2)} = \Theta_{\mu_1,\sigma_1} + \Theta_{\mu_2,\sigma_2} \tag{32}$$

$$\Theta_{(\mu_1\circ\mu_2)(\nu_1\circ\nu_2),(\sigma_1+\sigma_2)} = \Theta_{\mu_1\nu_1,\sigma_1} + \Theta_{\mu_2\nu_2,\sigma_2} \tag{33}$$

$$\Theta_{(\mu_1\circ\mu_2)(\nu_1\circ\nu_2)(\lambda_1\circ\lambda_2),(\sigma_1+\sigma_2)} = \Theta_{\mu_1\nu_1\lambda_1,\sigma_1} + \Theta_{\mu_2\nu_2\lambda_2,\sigma_2} \tag{34}$$

which means that all the topological invariants are linear under "vertical" fusions. We firstly prove (33) as the following: The the right-hand side of (33) is:

$$\begin{aligned} e^{i(\Theta_{\mu_1\nu_1,\sigma_1}+\Theta_{\mu_2\nu_2,\sigma_2})}I &= (B^{\delta_1}_{\xi^1_\mu\xi^1_\nu,e^1_\sigma})^{N^{\mu\nu}}(B^{\delta_2}_{\xi^2_\mu\xi^2_\nu,e^2_\sigma})^{N^{\mu\nu}} \\ &= (B^{\delta'}_{\xi'_\mu\xi'_\nu,(e^1_\sigma+e^2_\sigma)})^{N^{\mu\nu}} \end{aligned} \tag{35}$$

where we have applied the eqn.(30), and $B^{\delta'}_{\xi'_\mu\xi'_\nu,(e^1_\sigma+e^2_\sigma)}$ can be any of the diagonalized entry in the matrix after fusion (29) introduced above. The left-hand side of (33) is:

$$\begin{aligned} e^{i\Theta_{(\mu_1\circ\mu_2)(\nu_1\circ\nu_2),(\sigma_1+\sigma_2)}}I &= (B^{\delta''}_{(\xi^1_\mu\circ\xi^2_\mu)(\xi^1_\nu\circ\xi^2_\nu),(e^1_\sigma+e^2_\sigma)})^{N^{\mu\nu}} \\ &= (B^{\delta''}_{\xi''_\mu\xi''_\nu,(e^1_\sigma+e^2_\sigma)})^{N^{\mu\nu}} \end{aligned} \tag{36}$$

where $B^{\delta''}_{\xi_\mu\xi_\nu,(e^1_\sigma+e^2_\sigma)}$ can also be any of the entry in the same diagonalized matrix. Then the difference between $B^{\delta'}_{\xi'_\mu\xi'_\nu,(e^1_\sigma+e^2_\sigma)}$ and $B^{\delta''}_{\xi''_\mu\xi''_\nu,(e^1_\sigma+e^2_\sigma)}$ can be eliminiated by the $N^{\mu\nu}$ times of braiding, which is shown in eqn.(15) of Ref[1].

Then (32) and (34) can be proved similarly, as $\Theta_{\mu,\sigma}$ and $\Theta_{\mu\nu\lambda,\sigma}$ are also defined so as to eliminate the effect caused by difference charge attachments.

C. Partial Proof of the Constraints

We can rigorously prove the following constraints:

$$N_{\mu\nu\lambda\sigma}\Theta_{\mu\nu\lambda,\sigma} = 0 \tag{37}$$

$$N_\sigma\Theta_{\mu\nu,\sigma} = 0 \tag{38}$$

$$N_\sigma\Theta_{\mu,\sigma} = 0 \tag{39}$$

We firstly prove (38) as the following: By the property (33), we have:

$$\begin{aligned} e^{iN_\sigma\Theta_{\mu\nu,\sigma}}I &= e^{i(\Theta_{\mu_1\nu_1,\sigma_1}+\ldots+\Theta_{\mu_{N_\sigma}\nu_{N_\sigma},\sigma_{N_\sigma}})}I \\ &= e^{i\Theta_{(\mu_1\circ\ldots\circ\mu_{N_\sigma})(\nu_1\circ\ldots\circ\nu_{N_\sigma}),(\sigma_1+\ldots+\sigma_{N_\sigma})}}I \\ &= (B^\delta_{\xi_\mu\xi_\nu})^{N^{\mu\nu}} = I \end{aligned} \tag{40}$$

where we realize the phase $N_\sigma\Theta_{\mu\nu,\sigma}$ by constructing N_σ identical three-loop systems, and then applying the "vertical" fusions, by which the N_σ type-σ base loops all together vanish. And by (32) and (34), (37) and (39) can be proved similarly.

IV. SUPPLEMENTARY NOTE 4: SOLVING THE CONSTRAINTS

A. Category (A)

The constraints that are related to 2D constraints by dimension reduction are:

$$\Theta_{000,0} = m\Theta_{000,0} \tag{41}$$

$$N_0\Theta_{00,0} = \mathcal{F}(N_0)\Theta_{000,0} = m\Theta_{000,0} \tag{42}$$

$$\Theta_{00,0} = \begin{cases} 2\Theta_{0,0}, & \text{if } m \text{ is even} \\ 4\Theta_{0,0} + \Theta_{000,0}, & \text{if } m \text{ is odd} \end{cases} \tag{43}$$

$$\begin{cases} \frac{m}{2}\Theta_{0,0} = 0, & \text{if } m \text{ is even} \\ m\Theta_{0,0} + \frac{m^2-1}{8}\Theta_{000,0} = 0, & \text{if } m \text{ is odd} \end{cases} \tag{44}$$

The newly involved 3D constraints are:

$$2\Theta_{000,0} = 0 \Rightarrow \Theta_{000,0} = 0 \text{ or } \pi \tag{45}$$

$$\Theta_{0,0} = 0 \tag{46}$$

We solve the constraints in two cases:

1. m is odd

By $\Theta_{0,0}=0$ and the constraint $\Theta_{00,0}=4\Theta_{0,0}+\Theta_{000,0}$, we have $\Theta_{00,0}=\Theta_{000,0}$. Combining $\Theta_{00,0}=\Theta_{000,0}$ and $N_0\Theta_{00,0}=m\Theta_{000,0}$, we find $\Theta_{00,0}$ and $\Theta_{000,0}$ can only both be 0. Hence

$$(\Theta_{0,0},\Theta_{00,0},\Theta_{000,0})=(0,0,0) \tag{47}$$

The classification is trivial.

2. m is even

By $\Theta_{0,0}=0$ and the constraint $\Theta_{00,0}=2\Theta_{0,0}$, we have $\Theta_{00,0}=0$. And the constraint $\Theta_{000,0}=m\Theta_{000,0}$ ensures that $\Theta_{000,0}=0$. Hence

$$(\Theta_{0,0},\Theta_{00,0},\Theta_{000,0})=(0,0,0) \tag{48}$$

The classification is trivial.

B. Category (B)

For simplicity, we only consider symmetry groups with order being power of 2.

The newly involved 3D constraints are:

$$\Theta_{i,i}=0 \tag{49}$$

$$N_0\Theta_{i,0}=0,\quad N_i\Theta_{0,i}=0 \tag{50}$$

$$N_0\Theta_{0i,0}=0,\quad N_i\Theta_{0i,i}=0 \tag{51}$$

$$N_0\Theta_{00i,0}=0,\quad N_i\Theta_{00i,i}=0 \tag{52}$$

$$\Theta_{0i,i}=-\frac{N^{0i}}{\widetilde{N}_i}\Theta_{i,0},\quad \Theta_{0i,0}=-\frac{N^{0i}}{\widetilde{N}_0}\Theta_{0,i} \tag{53}$$

$$\Theta_{00i,i}=\Theta_{0ii,0}=\Theta_{00i,0}=\Theta_{000,i} \tag{54}$$

1. m is odd

For m is odd, we set $m=1$ for simplicity, i.e. we only consider the symmetry group $\mathbb{Z}_2\times\mathbb{Z}_{N_i}$. We can do this as $\mathbb{Z}_{2m}^f$ is isomorphic to $\mathbb{Z}_2^f\times\mathbb{Z}_m$, and $\mathbb{Z}_m$ can be absorbed into $G_b=\prod_i\mathbb{Z}_{N_i}$ part of G_f, making G_f always the form $G_f=\mathbb{Z}_2^f\times G_b$.

(1) If $\frac{N_i}{2}$ is odd (i.e. $\mathbb{Z}_2^f\times\mathbb{Z}_2$), invoking the known 2D results and combining with the 3D constraints $N_0\Theta_{i,0}=0$, $N_i\Theta_{0,i}=0$, $N_0\Theta_{0i,0}=0$, $N_i\Theta_{0i,i}=0$, $N_0\Theta_{00i,0}=0$, $N_i\Theta_{00i,i}=0$, the generating phases for the sets (B1), (B2), and (B3) are:

$$\Theta_{0,i}=\frac{2\pi}{m}=0 \tag{55}$$

$$\begin{aligned}(\Theta_{i,0},\Theta_{0i,0},\Theta_{00i,0})&=(\frac{\pi}{2N_i},-\frac{\pi}{N_{0i}},\pi)\times 2N_ia+(0,\frac{4\pi}{N_{0i}},0)\\&=(\pi,0,0)a\end{aligned} \tag{56}$$

$$\begin{aligned}(\Theta_{i,i},\Theta_{0i,i},\Theta_{00i,i})&=(\frac{\pi}{2N_i},\mp\frac{\pi}{N_{0i}},\pi)\times 0+(0,\frac{4\pi}{N_{0i}},0)\\&=(0,0,0)\end{aligned} \tag{57}$$

where a is an integer.

By the constraint $\Theta_{0i,i}=-\Theta_{i,0}$, $a=0$ (mod 2). Hence in this case the classification is trivial.

(2) If $N_i=4$ (mod 8), similarly, the generating phases for the sets (B1), (B2), and (B3) are:

$$\Theta_{0,i}=\frac{2\pi}{m}=0 \tag{58}$$

$$\begin{aligned}(\Theta_{i,0},\Theta_{0i,0},\Theta_{00i,0})&=(\frac{\pi}{N_i},\frac{2\pi}{N_{0i}},\pi)\times N_ia+(0,\frac{2\pi}{N_{0i}},\pi)\times b\\&=(\pi,0,0)a+(0,\pi,\pi)b\end{aligned} \tag{59}$$

$$\begin{aligned}(\Theta_{i,i},\Theta_{0i,i},\Theta_{00i,i})&=(\frac{\pi}{N_i},\frac{2\pi}{N_{0i}},\pi)\times 0+(0,\frac{2\pi}{N_{0i}},\pi)\times c\\&=(0,\pi,\pi)c\end{aligned} \tag{60}$$

where a,b,c are all integers.

By the constraint $\Theta_{0i,0}=-N_i\Theta_{0,i}$, $b=0$ (mod 2). By the constraint $\Theta_{00i,i}=\Theta_{00i,0}$, $c=0$ (mod 2). By the constraint $\Theta_{0i,i}=-\Theta_{i,0}$, $a=0$ (mod 2). Hence in this case the classification is trivial.

(3) If $N_i=0$ (mod 8), the generating phases for the sets (B1), (B2), and (B3) are:

$$\Theta_{0,i}=\frac{2\pi}{m}=0 \tag{61}$$

$$\begin{aligned}(\Theta_{i,0},\Theta_{0i,0},\Theta_{00i,0})&=(\frac{\pi}{N_i},\frac{2\pi}{N_{0i}},\pi)\times N_ia,(0,\frac{4\pi}{N_{0i}},\pi)\times b\\&=(\pi,0,0)a+(0,0,\pi)b\end{aligned} \tag{62}$$

$$\begin{aligned}(\Theta_{i,i},\Theta_{0i,i},\Theta_{00i,i})&=(\frac{\pi}{N_i},\frac{2\pi}{N_{0i}},\pi)\times 0+(0,\frac{4\pi}{N_{0i}},\pi)\times c\\&=(0,0,\pi)c\end{aligned} \tag{63}$$

By $\Theta_{0,i}=0$ and the constraint $\Theta_{00,i}=4\Theta_{0,i}+\Theta_{000,i}$, we have $\Theta_{00,i}=\Theta_{000,i}$. Combining $\Theta_{00,i}=\Theta_{000,i}$ and $N_0\Theta_{00,i}=m\Theta_{000,i}$, we find $\Theta_{00,0}=\Theta_{000,0}=0$. By $\Theta_{000,i}=0$ and the constraint $\Theta_{00i,i}=\Theta_{00i,0}=\Theta_{000,i}$, $b=c=0$ (mod 2). By the constraint $\Theta_{0i,i}=-\Theta_{i,0}$, $a=0$ (mod 2). Hence in this case the classification is trivial.

2. m is even

(1) If $N_i < N_0 \leq 4$ (i.e. $\mathbb{Z}_4^f \times \mathbb{Z}_2$), the generating phases for the sets (B1), (B2), and (B3) are:

$$\Theta_{0,i} = 0 \tag{64}$$

$$\begin{aligned}(\Theta_{i,0}, \Theta_{0i,0}, \Theta_{00i,0}) &= (\frac{\pi}{N_i}, \frac{2\pi}{N_{0i}}, 0) \times a + (0, \frac{4\pi}{N_{0i}}, 0) \\ &= (\frac{\pi}{2}, \pi, 0)a\end{aligned} \tag{65}$$

$$(\Theta_{i,i}, \Theta_{0i,i}, \Theta_{00i,i}) = (\frac{\pi}{N_i}, \frac{2\pi}{N_{0i}}, 0)\times 0 + (0, \frac{4\pi}{N_{0i}}, 0) = (0,0,0) \tag{66}$$

By the constraint $\Theta_{0i,0} = -\Theta_{0,i}$, $a = 0, 2 \pmod 4$. And the remaining generating phase is determined by integer a. Hence in this case the classification is $\mathbb{Z}_2$, which belongs to BSPT phases.

(2) If $N_i < 4 < N_0$ (i.e. $\mathbb{Z}_{N_0}^f \times \mathbb{Z}_2$), the generating phases for the sets (B1), (B2), and (B3) are:

$$\Theta_{0,i} = \frac{4\pi}{m} \times \frac{m}{4} a = \pi a \tag{67}$$

$$\begin{aligned}(\Theta_{i,0}, \Theta_{0i,0}, \Theta_{00i,0}) &= (\frac{\pi}{N_i}, \frac{2\pi}{N_{0i}}, 0) \times b + (0, \frac{4\pi}{N_{0i}}, 0) \times c \\ &= (\frac{\pi}{2}, \pi, 0)b\end{aligned} \tag{68}$$

$$(\Theta_{i,i}, \Theta_{0i,i}, \Theta_{00i,i}) = (\frac{\pi}{N_i}, \frac{2\pi}{N_{0i}}, 0)\times 0 + (0, \frac{4\pi}{N_{0i}}, 0) = (0,0,0) \tag{69}$$

By the constraint $\Theta_{0i,0} = -\Theta_{0,i}$, $b = -a \pmod 4$. By the constraint $\Theta_{0i,i} = -m\Theta_{i,0}$, $0 = -m\frac{\pi}{2}b$, which is always satisfied as in this case the smallest m is 4. The generating phases are generated by integer b. Hence in this case the classification is $\mathbb{Z}_4$, which is a $\mathbb{Z}_2$ complex fermion layer absorbed into a $\mathbb{Z}_2$ BSPT layer as the complex fermion layer indicator is $\Theta_{fi,j} = \Theta_{0i,j} = \pi b$.

(3) If $4 \leq N_i < N_0$, the generating phases for the sets (B1), (B2), and (B3) are:

$$\Theta_{0,i} = \begin{cases} \frac{4\pi}{m}a, & \text{if } N_i > N_0/4 \\ \frac{8\pi}{N_0} \times \frac{N_0}{4N_i}a = \frac{2\pi}{N_i}a, & \text{if } N_i \leq N_0/4 \end{cases} \tag{70}$$

$$\begin{aligned}(\Theta_{i,0}, \Theta_{0i,0}, \Theta_{00i,0}) &= (\frac{\pi}{N_i}, \frac{2\pi}{N_{0i}}, 0) \times b + (0, \frac{4\pi}{N_{0i}}, 0) \times c \\ &= (\frac{\pi}{N_i}b, \frac{2\pi}{N_i}b + \frac{4\pi}{N_i}c, 0)\end{aligned} \tag{71}$$

$$\begin{aligned}(\Theta_{i,i}, \Theta_{0i,i}, \Theta_{00i,i}) &= (\frac{\pi}{N_i}, \frac{2\pi}{N_{0i}}, 0) \times 0 + (0, \frac{4\pi}{N_{0i}}, 0) \times d \\ &= (0, \frac{4\pi}{N_i}d, 0)\end{aligned} \tag{72}$$

By the constraint $\Theta_{0i,0} = -\Theta_{0,i}$,

$$\begin{cases} \frac{2\pi}{N_i}b + \frac{4\pi}{N_i}c = -\frac{8\pi}{N_0}a, & \text{if } N_i > N_0/4 \\ \frac{2\pi}{N_i}(b+2c) = -\frac{2\pi}{N_i}a, & \text{if } N_i \leq N_0/4 \end{cases} \tag{73}$$

By the constraint $\Theta_{0i,i} = -\frac{N_0}{N_i}\Theta_{i,0}$, $\frac{4\pi}{N_i}d = -\frac{N_0}{N_i}\frac{\pi}{N_i}b$, we have:

$$\begin{cases} b = -\frac{4N_i}{N_0}d, & \text{if } N_i > N_0/4 \text{ and } N_0 < 2N_i^2 \\ d = -\frac{N_0}{4N_i}b, & \text{if } N_i \leq N_0/4 \end{cases} \tag{74}$$

Combining all the constraints:

$$\begin{cases} (b+2c) = -2a,\ b = -2d, & \text{if } N_i = N_0/2 \\ (b+2c) = -a,\ d = -\frac{N_0}{4N_i}b, & \text{if } N_i \leq N_0/4 \end{cases} \tag{75}$$

The generating phases are determined by the integers:

$$\begin{cases} a \pmod{N_0/4},\ b \pmod{\frac{1}{2} \times 2N_i = N_i}, & \text{if } N_i = N_0/2 \\ c \pmod{N_i/2},\ b \pmod{2N_i}, & \text{if } N_i \leq N_0/4 \end{cases} \tag{76}$$

Hence in this case the classification is:

$$\begin{cases} \mathbb{Z}_{N_i} \times \mathbb{Z}_{N_0/4} = \mathbb{Z}_{N_i} \times \mathbb{Z}_{N_i/2} \text{ (BSPT)}, & \text{if } N_i = N_0/2 \\ \mathbb{Z}_{2N_i} \times \mathbb{Z}_{N_i/2} \text{ (a } \mathbb{Z}_2 \text{ complex fermion layer absorbed into a } \mathbb{Z}_{N_i} \times \mathbb{Z}_{N_i/2} \text{ BSPT layer)}, & \text{if } N_i \leq N_0/4 \end{cases} \tag{77}$$

where the indicators of the complex fermion layer are:

$$\begin{cases} \Theta_{fi,0} = \frac{N_i}{2}\Theta_{0i,0} = -2\pi a,\ \Theta_{fi,i} = \frac{N_i}{2}\Theta_{0i,i} = 0, & \text{if } N_i = N_0/2 \\ \Theta_{fi,0} = \frac{N_i}{2}\Theta_{0i,0} = -\pi a,\ \Theta_{fi,i} = \frac{N_i}{2}\Theta_{0i,i} = 0, & \text{if } N_i \leq N_0/4 \end{cases} \tag{78}$$

(4) If $4 \leq N_0 \leq N_i$, the generating phases for the sets (B1), (B2), and (B3) are:

$$\Theta_{0,i} = \begin{cases} 0, & \text{if } N_0 = 4 \\ \frac{4\pi}{m}a, & \text{if } N_0 > 4 \end{cases} \tag{79}$$

$$(\Theta_{i,0},\Theta_{0i,0},\Theta_{00i,0})=(\frac{\pi}{N_i},\frac{2\pi}{N_{0i}},0)\times\frac{2N_i}{N_0}b+(0,\frac{4\pi}{N_{0i}},0)\times c=(\frac{2\pi}{N_0}b,\frac{4\pi}{N_0}(\frac{N_i}{N_0}b+c),0) \tag{80}$$

$$(\Theta_{i,i},\Theta_{0i,i},\Theta_{00i,i})=(\frac{\pi}{N_i},\frac{2\pi}{N_{0i}},0)\times 0+(0,\frac{4\pi}{N_{0i}},0)\times d=(0,\frac{4\pi}{N_0}d,0) \tag{81}$$

By the constraint $\Theta_{0i,0}=-\frac{N_i}{N_0}\Theta_{0,i}$, $\frac{4\pi}{N_0}(\frac{N_i}{N_0}b+c)=-\frac{N_i}{N_0}\frac{8\pi}{N_0}a$, where when $N_0=4$ or $N_i\geq N_0^2/4$, the right-hand side becomes 0 (mod 2π). Then we have:

$$\begin{cases}\frac{4\pi}{N_0}(\frac{N_i}{N_0}b+c)=0, & \text{if } N_0=4\\ \frac{4\pi}{N_0}(\frac{N_i}{N_0}b+c)=0, & \text{if } N_0>4 \text{ and } N_i\geq N_0^2/4\\ (\frac{N_i}{N_0}b+c)=-\frac{2N_i}{N_0}a, & \text{if } N_0>4 \text{ and } N_i< N_0^2/4\end{cases} \tag{82}$$

By the constraint $\Theta_{0i,i}=-\Theta_{i,0}$, $\frac{4\pi}{N_0}d=-\frac{2\pi}{N_0}b$, $b=-2d$.

The generating phases are determined by the integers:

$$\begin{cases} d \pmod{N_0/2}, & \text{if } N_0=4\\ a \pmod{N_0/4},\ d \pmod{N_0/2}, & \text{if } N_0>4\end{cases} \tag{83}$$

Hence in this case the classification is

$$\begin{cases}\mathbb{Z}_{N_0/2}=\mathbb{Z}_2 \ \text{(BSPT)}, & \text{if } N_0=4\\ \mathbb{Z}_{N_0/4}\times\mathbb{Z}_{N_0/2}\ \text{(BSPT)}, & \text{if } N_0>4\end{cases} \tag{84}$$

where the indicators of the complex fermion layer are:

$$\begin{cases}\Theta_{fi,0}=\frac{N_0}{2}\Theta_{0i,0}=0,\ \Theta_{fi,i}=\frac{N_0}{2}\frac{4\pi}{N_0}d=0, & \text{if } N_0=4\\ \Theta_{fi,0}=\frac{N_0}{2}\Theta_{0i,0}=0,\ \Theta_{fi,i}=\frac{N_0}{2}\Theta_{0i,i}=0, & \text{if } N_0>4\end{cases} \tag{85}$$

For m is even, combining the cases (1)(2) into (3)(4), in conclusion the classification is:

$$\begin{cases}\mathbb{Z}_{N_i}\times\mathbb{Z}_{N_i/2}, & \text{if } N_i=N_0/2\\ \mathbb{Z}_{2N_i}\times\mathbb{Z}_{N_i/2}, & \text{if } N_i<N_0/2\\ \mathbb{Z}_{N_0/2}\times\mathbb{Z}_{N_0/4}, & \text{if } N_0\leq N_i\end{cases} \tag{86}$$

which means that the BSPT classification is $\mathbb{Z}_{\min\{N_i,N_0/2\}}\times\mathbb{Z}_{\min\{N_i,N_0/2\}/2}$, and a $\mathbb{Z}_2$ complex fermion layer will be absorbed in the BSPT layer when $N_i<N_0/2$.

C. Category (C)

For simplicity, we only consider symmetry groups with order being power of 2. The newly involved constraints in 3D are:

$$N_\sigma\Theta_{\mu,\sigma}=0 \tag{87}$$

$$N_\sigma\Theta_{\mu\nu,\sigma}=0 \tag{88}$$

$$N_\sigma\Theta_{\mu\nu\lambda,\sigma}=0 \tag{89}$$

$$\frac{N^{0ij}}{N^{ij}}\Theta_{ij,0}+\frac{N^{0ij}}{N^{0j}}\Theta_{0j,i}+\frac{N^{0ij}}{N^{0i}}\Theta_{0i,j}=0 \tag{90}$$

$$\Theta_{ij,i}=-\frac{N^{ij}}{\widetilde{N}_i}\Theta_{i,j},\quad \Theta_{ij,j}=-\frac{N^{ij}}{\widetilde{N}_j}\Theta_{j,i} \tag{91}$$

$$\Theta_{0ij,0}=\Theta_{00i,j}=\Theta_{0ii,j}=-\Theta_{0ij,i}=-\Theta_{00j,i}=-\Theta_{0jj,i}=-\Theta_{0ij,j} \tag{92}$$

1. m is odd

Similarly we also set $m=1$, so that we only need to consider the symmetry group $\mathbb{Z}_2\times\mathbb{Z}_{N_i}\times\mathbb{Z}_{N_j}$, and we assume $N_i\le N_j$ without loss of generality.

(1) If $\frac{N_i}{2},\frac{N_j}{2}$ are odd (i.e. $\mathbb{Z}_2^f\times\mathbb{Z}_2\times\mathbb{Z}_2$), invoking the known 2D results and combining with the 3D constraints $N_\sigma\Theta_{\mu,\sigma}=0$, $N_\sigma\Theta_{\mu\nu,\sigma}=0$, $N_\sigma\Theta_{\mu\nu\lambda,\sigma}=0$, the generating phases for the sets (C1), (C2), (C3), (C4) and (C5) are:

$$(\Theta_{ij,0},\Theta_{0ij,0})=(\frac{\pi}{N_{ij}},\frac{2\pi}{N_{0ij}})\times 2a+(0,\frac{4\pi}{N_{0ij}})=(\pi a,0) \tag{93}$$

$$(\Theta_{ij,i},\Theta_{0ij,i})=(\frac{\pi}{N_{ij}},\frac{2\pi}{N_{0ij}})\times 2b+(0,\frac{4\pi}{N_{0ij}})=(\pi b,0) \tag{94}$$

$$(\Theta_{ij,j},\Theta_{0ij,j})=(\frac{\pi}{N_{ij}},\frac{2\pi}{N_{0ij}})\times 2c+(0,\frac{4\pi}{N_{0ij}})=(\pi c,0) \tag{95}$$

$$(\Theta_{i,j},\Theta_{0i,j},\Theta_{00i,j})=(\frac{\pi}{2N_i},-\frac{\pi}{N_{0i}},\pi)\times 4d+(0,\frac{4\pi}{N_{0i}},0)=(\pi,0,0)d \tag{96}$$

$$(\Theta_{j,i},\Theta_{0j,i},\Theta_{00j,i})=(\frac{\pi}{2N_j},-\frac{\pi}{N_{0j}},\pi)\times 4e+(0,\frac{4\pi}{N_{0j}},0)=(\pi,0,0)e \tag{97}$$

where a,b,c,d,e are integers.

By the constraint $\Theta_{ij,0}+\Theta_{oj,i}+\Theta_{0i,j}=0$, $a=0$ (mod 2).

By the constraint $\Theta_{ij,i}=-\Theta_{i,j}$, $b=-d$ (mod 2).

By the constraint $\Theta_{ij,j}=-\Theta_{j,i}$, $c=-e$ (mod 2).

Hence in this case the classification is $\mathbb{Z}_2\times\mathbb{Z}_2$, which belongs to BSPT.

(2) If $\frac{N_i}{2}$ is odd and $\frac{N_j}{2}$ is even (i.e. $\mathbb{Z}_2^f\times\mathbb{Z}_2\times\mathbb{Z}_{N_j}$), the generating phases for the sets (C1), (C2), (C3), (C4) and (C5) are:

$$(\Theta_{ij,0},\Theta_{0ij,0})=(\frac{2\pi}{N_{ij}},0)\times a+(0,\frac{2\pi}{N_{0ij}})\times b=(\pi a,\pi b) \tag{98}$$

$$(\Theta_{ij,i},\Theta_{0ij,i})=(\frac{2\pi}{N_{ij}},0)\times c+(0,\frac{2\pi}{N_{0ij}})\times d=(\pi c,\pi d) \tag{99}$$

$$(\Theta_{ij,j},\Theta_{0ij,j})=(\frac{2\pi}{N_{ij}},0)\times e+(0,\frac{2\pi}{N_{0ij}})\times f=(\pi e,\pi f) \tag{100}$$

$$(\Theta_{i,j},\Theta_{0i,j},\Theta_{00i,j})=\begin{cases}(\frac{\pi}{2N_i},-\frac{\pi}{N_{0i}},\pi)\times 2g+(0,\frac{4\pi}{N_{0i}},0)=(\frac{\pi}{2},-\pi,0)g, & \text{if } N_j=4\\ (\frac{\pi}{2N_i},-\frac{\pi}{N_{0i}},\pi)\times g+(0,\frac{4\pi}{N_{0i}},0)=(\frac{\pi}{4},-\frac{\pi}{2},\pi)g, & \text{if } N_j>4\end{cases} \tag{101}$$

$$(\Theta_{j,i},\Theta_{0j,i},\Theta_{00j,i})=\begin{cases}(\frac{\pi}{N_j},\frac{2\pi}{N_{0j}},0)\times 4h+(0,\frac{2\pi}{N_{0j}},\pi)\times i=(\pi h,\pi i,\pi i), & \text{if } N_j=4\\ (\frac{\pi}{N_j},\frac{2\pi}{N_{0j}},0)\times N_jh+(0,\frac{4\pi}{N_{0j}},\pi)\times i=(\pi h,0,\pi i), & \text{if } N_j>4\end{cases} \tag{102}$$

By the constraint $\Theta_{ij,0}+\Theta_{oj,i}+\frac{N_j}{2}\Theta_{0i,j}=0$,

$$\begin{cases}\pi a+\pi i=0,\ a=i \text{ (mod 2)}, & \text{if } N_j=4\\ \pi a+\frac{N_j}{2}(-\frac{\pi}{2}g)=0,\ a=0 \text{ (mod 2)}, & \text{if } N_j>4\end{cases} \tag{103}$$

By the constraint $\Theta_{ij,i} = -\frac{N_j}{2}\Theta_{i,j}$,

$$\begin{cases} \pi c = -\pi g,\ c = -g \ (\text{mod } 4), & \text{if } N_j = 4 \\ \pi c = -\frac{N_j}{2}(\frac{\pi}{4}g),\ c = -g \ (\text{mod } 8), & \text{if } N_j = 8 \\ \pi c = -\frac{N_j}{2}(\frac{\pi}{4}g),\ c = 0 \ (\text{mod } 2), & \text{if } N_j > 8 \end{cases} \tag{104}$$

By the constraint $\Theta_{ij,j} = -\Theta_{j,i}$, $\pi e = -\pi h$, $e = -h$ (mod 2).
By the constraint $\Theta_{0ij,0} = \Theta_{00i,j} = -\Theta_{0ij,i} = -\Theta_{00j,i} = -\Theta_{0ij,j}$,

$$\begin{cases} b = d = f = i = 0 \ (\text{mod } 2), & \text{if } N_j = 4 \\ b = d = f = g = i \ (\text{mod } 2), & \text{if } N_j > 4 \end{cases} \tag{105}$$

Combining all the constraints:

$$\begin{cases} a = b = d = f = i = 0 \ (\text{mod } 2),\ c = -g \ (\text{mod } 4),\ e = -h \ (\text{mod } 2), & \text{if } N_j = 4 \\ a = 0 \ (\text{mod } 2),\ b = d = f = g = i = -c \ (\text{mod } 8),\ e = -h \ (\text{mod } 2), & \text{if } N_j = 8 \\ a = 0 \ (\text{mod } 2), b = d = f = g = i \ (\text{mod } 8),\ c = 0 \ (\text{mod } 2),\ e = -h \ (\text{mod } 2), & \text{if } N_j > 8 \end{cases} \tag{106}$$

Hence in this case the classification is

$$\begin{cases} \mathbb{Z}_4 \times \mathbb{Z}_2 \ (\text{a } \mathbb{Z}_2 \text{ complex fermion layer aborbed into a } \mathbb{Z}_2 \times \mathbb{Z}_2 \text{ BSPT layer}), & \text{if } N_i = 2,\ N_j = 4 \\ \mathbb{Z}_8 \times \mathbb{Z}_2 \ (\text{a } \mathbb{Z}_2 \text{ Kitaev-chain layer further aborbed into the } \mathbb{Z}_4 \times \mathbb{Z}_2 \text{ above}), & \text{if } N_i = 2,\ N_j > 4 \end{cases} \tag{107}$$

where the indicator of the complex fermion layer is:

$$\begin{cases} \Theta_{fi,j} = \Theta_{0i,j} = -\pi g, & \text{if } N_i = 2,\ N_j = 4 \\ \Theta_{fi,j} = \Theta_{0i,j} = -\frac{\pi}{2}g, & \text{if } N_i = 2,\ N_j > 4 \end{cases} \tag{108}$$

(3) If $\frac{N_i}{2}, \frac{N_j}{2}$ are even (i.e. $\mathbb{Z}_2^f \times \mathbb{Z}_{N_i} \times \mathbb{Z}_{N_j}$) and let $N_i \le N_j$ without loss of generality, the generating phases for the sets (C1), (C2), (C3), (C4) and (C5) are:

$$(\Theta_{ij,0}, \Theta_{0ij,0}) = (\frac{2\pi}{N_{ij}}, 0) \times \frac{N_{ij}}{2}a + (0, \frac{2\pi}{N_{0ij}}) \times \frac{N_{0ij}}{2}b = (\pi a, \pi b) \tag{109}$$

$$(\Theta_{ij,i}, \Theta_{0ij,i}) = (\frac{2\pi}{N_{ij}}, 0) \times c + (0, \frac{2\pi}{N_{0ij}}) \times d = (\frac{2\pi}{N_i}c, \pi d) \tag{110}$$

$$(\Theta_{ij,j}, \Theta_{0ij,j}) = (\frac{2\pi}{N_{ij}}, 0) \times e + (0, \frac{2\pi}{N_{0ij}}) \times f = (\frac{2\pi}{N_i}e, \pi f) \tag{111}$$

$$(\Theta_{i,j}, \Theta_{0i,j}, \Theta_{00i,j}) = \begin{cases} (\frac{\pi}{N_i}, \frac{2\pi}{N_{0i}}, 0) \times 2g + (0, \frac{2\pi}{N_{0i}}, \pi) \times h = (\frac{\pi}{2}g, \pi h, \pi h), & \text{if } N_i = N_j = 4 \\ (\frac{\pi}{N_i}, \frac{2\pi}{N_{0i}}, 0) \times g + (0, \frac{2\pi}{N_{0i}}, \pi) \times h = (\frac{\pi}{4}g, \pi(g+h), \pi h), & \text{if } N_i = 4, N_j = 8 \\ (\frac{\pi}{N_i}, \frac{2\pi}{N_{0i}}, 0) \times 2g + (0, \frac{4\pi}{N_{0i}}, \pi) \times h = (\frac{2\pi}{N_i}g, 0, \pi h), & \text{if } 8 \le N_i = N_j \\ (\frac{\pi}{N_i}, \frac{2\pi}{N_{0i}}, 0) \times g + (0, \frac{4\pi}{N_{0i}}, \pi) \times h = (\frac{\pi}{N_i}g, \pi g, \pi h), & \text{if } 8 \le N_i < N_j \end{cases} \tag{112}$$

$$(\Theta_{j,i}, \Theta_{0j,i}, \Theta_{00j,i}) = \begin{cases} (\frac{\pi}{N_j}, \frac{2\pi}{N_{0j}}, 0) \times 2l + (0, \frac{2\pi}{N_{0j}}, \pi) \times m = (\frac{\pi}{2}l, \pi m, \pi m), & \text{if } N_i = N_j = 4 \\ (\frac{\pi}{N_j}, \frac{2\pi}{N_{0j}}, 0) \times 4l + (0, \frac{4\pi}{N_{0j}}, \pi) \times m = (\frac{\pi}{2}l, 0, \pi m), & \text{if } N_i = 4,\ N_j = 8 \\ (\frac{\pi}{N_j}, \frac{2\pi}{N_{0j}}, 0) \times 2l + (0, \frac{4\pi}{N_{0j}}, \pi) \times m = (\frac{2\pi}{N_j}l, 0, \pi m), & \text{if } 8 \le N_i = N_j \\ (\frac{\pi}{N_j}, \frac{2\pi}{N_{0j}}, 0) \times \frac{2N_j}{N_i}l + (0, \frac{4\pi}{N_{0j}}, \pi) \times m = (\frac{2\pi}{N_i}l, 0, \pi m), & \text{if } 8 \le N_i < N_j \end{cases} \tag{113}$$

By the constraint $\Theta_{ij,0} + \Theta_{oj,i} + \frac{Nj}{N_i}\Theta_{0i,j} = 0$,

$$\begin{cases} \pi a + \pi m + \pi h = 0, & \text{if } N_i = N_j = 4 \\ \pi a + \pi(g+h) = 0, & \text{if } N_i = 4, N_j = 8 \\ \pi a = 0, & \text{if } 8 \le N_i = N_j \\ \pi a + \pi g = 0, & \text{if } 8 \le N_i < N_j \end{cases} \tag{114}$$

By the constraint $\Theta_{ij,i} = -\frac{Nj}{N_i}\Theta_{i,j}$,

$$\begin{cases} c = -g, & \text{if } N_i = N_j = 4 \\ c = -g, & \text{if } N_i = 4, N_j = 8 \\ c = -g, & \text{if } 8 \le N_i = N_j \\ \frac{2\pi}{N_i}c = 0, & \text{if } 8 \le N_i < N_j \text{ and } N_j \ge 2N_i^2 \\ c = -\frac{Nj}{2N_i}g, & \text{if } 8 \le N_i < N_j \text{ and } N_j < 2N_i^2 \end{cases} \tag{115}$$

By the constraint $\Theta_{ij,j} = -\Theta_{j,i}$, $e = -l$.

By the constraint $\Theta_{0ij,0} = \Theta_{00i,j} = -\Theta_{0ij,i} = -\Theta_{00j,i} = -\Theta_{0ij,j}$, $b = d = f = h = m$.

Combine all the constraints:

$$\begin{cases} a = 0,\ b = d = f = h = m \pmod 2,\ c = -g \pmod 4,\ e = -l \pmod 4, & \text{if } N_i = N_j = 4 \\ a = g + h,\ b = d = f = h = m \pmod 2,\ c = -g \pmod 8,\ e = -l \pmod 4, & \text{if } N_i = 4, N_j = 8 \\ a = 0,\ b = d = f = h = m \pmod 2,\ c = -g \pmod{N_i},\ e = -l \pmod{N_i}, & \text{if } 8 \le N_i = N_j \\ a = g \pmod{2N_i},\ b = d = f = h = m \pmod 2,\ c = 0,\ e = -l \pmod{N_i}, & \text{if } 8 \le N_i < N_j \text{ and } N_j \ge 2N_i^2 \\ a = g \pmod{2N_i},\ b = d = f = h = m \pmod 2,\ c = -\frac{Nj}{2N_i}g,\ e = -l \pmod{N_i}, & \text{if } 8 \le N_i < N_j \text{ and } N_j < 2N_i^2 \end{cases} \tag{116}$$

Hence in this case the classification is

$$\begin{cases} \mathbb{Z}_4 \times \mathbb{Z}_4 \times \mathbb{Z}_2 \text{ (a } \mathbb{Z}_4 \times \mathbb{Z}_4 \text{ BSPT, stacking with a } \mathbb{Z}_2 \text{ "Kitaev-chain layer absorbed in complex fermion layer")}, \\ \text{if } N_i = N_j = 4 \\ \mathbb{Z}_8 \times \mathbb{Z}_4 \times \mathbb{Z}_2 \text{ (a } \mathbb{Z}_2 \text{ "complex fermion layer abrobed in a } \mathbb{Z}_4 \times \mathbb{Z}_4 \text{ BSPT"}, \\ \text{stacking with a } \mathbb{Z}_2 \text{ "Kitaev-chain layer absorbed in complex fermion layer")}, \quad \text{if } N_i = 4,\ N_j = 8 \\ \mathbb{Z}_{N_i} \times \mathbb{Z}_{N_i} \times \mathbb{Z}_2 \text{ (a } \mathbb{Z}_{N_i} \times \mathbb{Z}_{N_i} \text{ BSPT, stacking with a } \mathbb{Z}_2 \text{ Kitaev-chain layer)}, \quad \text{if } 8 \le N_i = N_j \\ \mathbb{Z}_{2N_i} \times \mathbb{Z}_{N_i} \times \mathbb{Z}_2 \text{ (a } \mathbb{Z}_2 \text{ complex fermion layer abrobed in } \mathbb{Z}_{N_i} \times \mathbb{Z}_{N_i} \text{ BSPT, stacking with a } \mathbb{Z}_2 \text{ Kitaev-chain layer)}, \\ \text{if } 8 \le N_i < N_j \end{cases} \tag{117}$$

where the complex fermion layer indicators are $\Theta_{fi,j} = \Theta_{0i,j}$ and $\Theta_{fj,i} = \Theta_{0j,i}$. And the classification can be simplified as:

$$\begin{cases} \mathbb{Z}_{N_i} \times \mathbb{Z}_{Ni} \times \mathbb{Z}_2, & \text{if } N_i = N_j \\ \mathbb{Z}_{2N_i} \times \mathbb{Z}_{Ni} \times \mathbb{Z}_2, & \text{if } N_i \ne N_j \end{cases} \tag{118}$$

2. m is even

By the constraint $m\Theta_{00i,j} = \Theta_{00i,j}$, $m\Theta_{00j,i} = \Theta_{00j,i}$, we have $\Theta_{00i,j} = 0$, $\Theta_{00j,i} = 0$.

By the constraint $\Theta_{0ij,0} = \Theta_{00i,j} = -\Theta_{0ij,i} = -\Theta_{00j,i} = -\Theta_{0ij,j}$, we have $\Theta_{0ij,0} = \Theta_{0ij,i} = \Theta_{0ij,j} = 0$, which means that there is no non-Abelian statistics in this case.

(1) If $\frac{N_i}{2}$, $\frac{N_j}{2}$ are odd (i.e. $\mathbb{Z}_{N_0}^f \times \mathbb{Z}_2 \times \mathbb{Z}_2$), the generating phases for the sets (C1), (C2), (C3), (C4) and (C5) are:

$$(\Theta_{ij,0}, \Theta_{0ij,0}) = (\frac{2\pi}{N_{ij}}, 0) \times a + (0, \frac{2\pi}{N_{0ij}}) \times b = (\pi a, \pi b) \tag{119}$$

$$(\Theta_{ij,i}, \Theta_{0ij,i}) = (\frac{2\pi}{N_{ij}}, 0) \times c + (0, \frac{2\pi}{N_{0ij}}) \times d = (\pi c, \pi d) \tag{120}$$

$$(\Theta_{ij,j}, \Theta_{0ij,j}) = (\frac{2\pi}{N_{ij}}, 0) \times e + (0, \frac{2\pi}{N_{0ij}}) \times f = (\pi e, \pi f) \tag{121}$$

$$(\Theta_{i,j}, \Theta_{0i,j}, \Theta_{00i,j}) = (\frac{\pi}{N_i}, \frac{2\pi}{N_{0i}}, 0) \times 2g + (0, \frac{4\pi}{N_{0i}}, 0) = (\pi, 0, 0)g \tag{122}$$

$$(\Theta_{j,i}, \Theta_{0j,i}, \Theta_{00j,i}) = (\frac{\pi}{N_j}, \frac{2\pi}{N_{0j}}, 0) \times 2h + (0, \frac{4\pi}{N_{0j}}, 0) = (\pi, 0, 0)h \tag{123}$$

By the constraint $\Theta_{0ij,0}=\Theta_{0ij,i}=\Theta_{0ij,j}=\Theta_{00i,j}=\Theta_{00j,i}=0$, $b=d=f=0$ (mod 2).

By the constraint $\frac{N_0}{2}\Theta_{ij,0}+\Theta_{oj,i}+\Theta_{0i,j}=0$, $\frac{N_0}{2}\pi a=0$, which is always satisfied.

By the constraint $\Theta_{ij,i}=-\Theta_{i,j}$, $c=-g$ (mod 2).

By the constraint $\Theta_{ij,j}=-\Theta_{j,i}$, $e=-h$ (mod 2).

The generating phases are determined by integers a (mod 2), g (mod 2), h (mod 2). And the classification is $\mathbb{Z}_2\times\mathbb{Z}_2\times\mathbb{Z}_2$ (BSPT).

(2) If $\frac{N_i}{2}$ is odd and $\frac{N_j}{2}$ is even (i.e. $\mathbb{Z}_{N_0}^f\times\mathbb{Z}_2\times\mathbb{Z}_{N_j}$), the generating phases for the sets (C1), (C2), (C3), (C4) and (C5) are:

$$(\Theta_{ij,0},\Theta_{0ij,0})=(\frac{2\pi}{N_{ij}},0)\times a+(0,\frac{2\pi}{N_{0ij}})\times 2b=(\pi a,0) \tag{124}$$

$$(\Theta_{ij,i},\Theta_{0ij,i})=(\frac{2\pi}{N_{ij}},0)\times c+(0,\frac{2\pi}{N_{0ij}})\times 2d=(\pi c,0) \tag{125}$$

$$(\Theta_{ij,j},\Theta_{0ij,j})=(\frac{2\pi}{N_{ij}},0)\times e+(0,\frac{2\pi}{N_{0ij}})\times 2f=(\pi e,0) \tag{126}$$

$$(\Theta_{i,j},\Theta_{0i,j},\Theta_{00i,j})=(\frac{\pi}{N_i},\frac{2\pi}{N_{0i}},0)\times g+(0,\frac{4\pi}{N_{0i}},0)\times h=(\frac{\pi}{2},\pi,0)g \tag{127}$$

$$(\Theta_{j,i},\Theta_{0j,i},\Theta_{00j,i})=(\frac{\pi}{N_j},\frac{2\pi}{N_{0j}},0)\times N_j l+(0,\frac{4\pi}{N_{0j}},0)\times\frac{N_{0j}}{4}m=(\pi l,\pi m,0) \tag{128}$$

By the constraint

$$\begin{cases}\Theta_{ij,0}+\Theta_{oj,i}+\frac{N_j}{N_0}\Theta_{0i,j}=0,\ \pi a+\pi m+\frac{N_j}{N_0}(\pi g)=0,\ a=m \text{ (mod 2)}, & \text{if } N_0<N_j\\ \Theta_{ij,0}+\Theta_{oj,i}+\Theta_{0i,j}=0,\ \pi a+\pi m+\pi g=0, & \text{if } N_0=N_j\\ \frac{N_0}{N_j}\Theta_{ij,0}+\Theta_{oj,i}+\Theta_{0i,j}=0,\ \frac{N_0}{N_j}\pi a+\pi m+\pi g=0,\ m=g \text{ (mod 4)} & \text{if } N_j<N_0\end{cases} \tag{129}$$

where g is chosen as a generating phase, only a,m need to be considered here.

By the constraint $\Theta_{ij,i}=-\frac{N_j}{2}\Theta_{i,j}$, $\pi c=-\frac{N_j}{2}(\frac{\pi}{2}g)$,

$$\begin{cases}\pi c=-\pi g,\ c=g \text{ (mod 4)}, & \text{if } N_j=4\\ \pi c=0,\ c=0 \text{ (mod 2)}, & \text{if } N_j>4\end{cases} \tag{130}$$

By the constraint $\Theta_{ij,j}=-\Theta_{j,i}$, $\pi e=-\pi l$, $e=l$ (mod 2).

Combine all the constraints:

$$\begin{cases} a=m \text{ (mod 2)},\ g \text{ (mod 4)},\ c=0 \text{ (mod 2)}, e=l \text{(mod 2)}, & \text{if } N_0<N_j\\ a \text{ (mod 2)},\ c=g \text{ (mod 4)},\ e=l \text{ (mod 2)}, & \text{if } 4=N_j=N_0\\ a \text{ (mod 2)}, g=m=c \text{(mod 4)},\ e=l \text{ (mod 2)}, & \text{if } 4=N_j<N_0\\ a \text{ (mod 2)},\ g \text{ (mod 4)},\ c=0 \text{ (mod 2)},\ e=l \text{ (mod 2)},, & \text{if } 4<N_j=N_0\\ a \text{ (mod 2)},\ g=m \text{ (mod 4)},\ c=0 \text{ (mod 2)},\ e=l \text{ (mod 2)}, & \text{if } 4<N_j<N_0\end{cases} \tag{131}$$

where the generating phases are determined by:

$$a \text{ (mod 2)},\ g \text{ (mod 4)},\ l \text{ (mod 2)},\quad \text{for all cases} \tag{132}$$

Hence in this case the classification is $\mathbb{Z}_4\times\mathbb{Z}_2\times\mathbb{Z}_2$ (a $\mathbb{Z}_2$ complex fermion layer absorbed into a $\mathbb{Z}_2\times\mathbb{Z}_2\times\mathbb{Z}_2$ BSPT layer), and the complex layer indicators are:

$$\Theta_{fi,j}=\Theta_{0i,j}=-\pi g,\ \Theta_{fj,i}=\frac{N_0}{2}\Theta_{0j,i}=0,\quad \text{for all cases} \tag{133}$$

(3) If $\frac{N_i}{2}$, $\frac{N_j}{2}$ are even (i.e. $\mathbb{Z}_{N_0}^f\times\mathbb{Z}_{N_i}\times\mathbb{Z}_{N_j}$) and $N_i=N_j$, the generating phases for the sets (C1), (C2), (C3), (C4) and (C5) are:

$$(\Theta_{ij,0},\Theta_{0ij,0})=\begin{cases}(\frac{2\pi}{N_{ij}},0)\times a,(0,\frac{2\pi}{N_{0ij}})\times N_{0ij}b=(\frac{2\pi}{N_i}a,0), & \text{if } N_0\geq N_i\\ (\frac{2\pi}{N_{ij}},0)\times\frac{N_{ij}}{N_0}a,(0,\frac{2\pi}{N_{0ij}})\times N_{0ij}b=(\frac{2\pi}{N_0}a,0), & \text{if } N_0\leq N_i\end{cases} \tag{134}$$

which can be simplified as (as N_0, N_i are powers of 2, $N_{0i} = \min\{N_0, N_i\}$):

$$(\Theta_{ij,0}, \Theta_{0ij,0}) = (\frac{2\pi}{N_{0i}}a, 0) \tag{135}$$

$$(\Theta_{ij,i}, \Theta_{0ij,i}) = (\frac{2\pi}{N_{ij}}, 0) \times c + (0, \frac{2\pi}{N_{0ij}}) \times N_{0ij}d = (\frac{2\pi}{N_i}c, 0) \tag{136}$$

$$(\Theta_{ij,j}, \Theta_{0ij,j}) = (\frac{2\pi}{N_{ij}}, 0) \times e + (0, \frac{2\pi}{N_{0ij}}) \times N_{0ij}f = (\frac{2\pi}{N_i}e, 0) \tag{137}$$

$$\Theta_{i,j}, \Theta_{0i,j}, \Theta_{00i,j}) = (\frac{\pi}{N_i}, \frac{2\pi}{N_{0i}}, 0) \times 2g + (0, \frac{4\pi}{N_{0i}}, 0) \times h = (\frac{2\pi}{N_i}g, \frac{4\pi}{N_{0i}}g + \frac{4\pi}{N_{0i}}h, 0) \tag{138}$$

$$(\Theta_{j,i}, \Theta_{0j,i}, \Theta_{00j,i}) = (\frac{\pi}{N_i}, \frac{2\pi}{N_{0i}}, 0) \times 2l + (0, \frac{4\pi}{N_{0i}}, 0) \times m = (\frac{2\pi}{N_i}l, \frac{4\pi}{N_{0i}}l + \frac{4\pi}{N_{0i}}m, 0) \tag{139}$$

By the constraint:

$$\begin{cases} \Theta_{ij,0} + \Theta_{oj,i} + \Theta_{0i,j} = 0, \ \frac{2\pi}{N_{0i}}a + \frac{4\pi}{N_{0i}}l + \frac{4\pi}{N_{0i}}m + \frac{4\pi}{N_{0i}}g + \frac{4\pi}{N_{0i}}h = 0, & \text{if } N_0 \le N_i \text{ and } N_0/2 < N_i \\ \frac{N_0}{N_i}\Theta_{ij,0} + \Theta_{oj,i} + \Theta_{0i,j} = 0, \frac{4\pi}{N_i}a + \frac{4\pi}{N_{0i}}l + \frac{4\pi}{N_{0i}}m + \frac{4\pi}{N_{0i}}g + \frac{4\pi}{N_{0i}}h = 0, & \text{if } N_0 > N_i \text{ and } N_0/2 = N_i \\ \frac{N_0}{N_i}\Theta_{ij,0} + \Theta_{oj,i} + \Theta_{0i,j} = 0, \frac{N_0}{N_i}(\frac{2\pi}{N_i}a) + \frac{4\pi}{N_{0i}}l + \frac{4\pi}{N_{0i}}m + \frac{4\pi}{N_{0i}}g + \frac{4\pi}{N_{0i}}h = 0, & \text{if } N_0 > N_i \text{ and } N_0/2 > N_i \end{cases} \tag{140}$$

where g, l are chosen as generating phases, only a, h, m need to be considered here.

By the constraint $\Theta_{ij,i} = -\frac{N_j}{2}\Theta_{i,j}$, $\frac{2\pi}{N_i}c = \pi g$, where the solution is:

$$c = 0, \frac{N_i}{2} \pmod{N_i} \text{ depending on } g \pmod{N_i} \tag{141}$$

By the constraint $\Theta_{ij,j} = -\Theta_{j,i}$, $\frac{2\pi}{N_i}e = -\frac{2\pi}{N_i}l$, $e = -l \pmod{N_i}$.

Combine all the constraints, the generating phases are:

$$\begin{cases} g \pmod{N_i}, \ l \pmod{N_i}, \ a \text{ or } m \pmod{N_0/2}, \ h \pmod{N_{0i}/2}, & \text{if if } N_0 \le N_i \text{ and } N_0/2 < N_i \\ g \pmod{N_i}, \ l \pmod{N_i}, \ a \pmod{N_i}, \ h \pmod{N_{0i}/2}, & \text{if } N_0 > N_i \text{ and } N_0/2 = N_i \\ g \pmod{N_i}, \ l \pmod{N_i}, \ a \pmod{N_i}, \ h \pmod{N_{0i}/2}, & \text{if } N_0 > N_i \text{ and } N_0/2 > N_i \end{cases} \tag{142}$$

Hence the classification is $\mathbb{Z}_{N_i} \times \mathbb{Z}_{N_i} \times \mathbb{Z}_{\min\{N_0/2, N_i\}} \times \mathbb{Z}_{N_{0i}/2}$ (BSPT).

(4) If $\frac{N_i}{2}, \frac{N_j}{2}$ are even (i.e. $\mathbb{Z}_{N_0}^f \times \mathbb{Z}_{N_i} \times \mathbb{Z}_{N_j}$) and $N_i < N_j$, the generating phases for the sets (C1), (C2), (C3), (C4) and (C5) are:

$$(\Theta_{ij,0}, \Theta_{0ij,0}) = (\frac{2\pi}{N_{0i}}a, 0) \tag{143}$$

$$(\Theta_{ij,i}, \Theta_{0ij,i}) = (\frac{2\pi}{N_{ij}}, 0) \times c + (0, \frac{2\pi}{N_{0ij}}) \times N_{0ij}d = (\frac{2\pi}{N_i}c, 0) \tag{144}$$

$$(\Theta_{ij,j}, \Theta_{0ij,j}) = (\frac{2\pi}{N_{ij}}, 0) \times e + (0, \frac{2\pi}{N_{0ij}}) \times N_{0ij}f = (\frac{2\pi}{N_i}e, 0) \tag{145}$$

$$(\Theta_{i,j}, \Theta_{0i,j}, \Theta_{00i,j}) = (\frac{\pi}{N_i}, \frac{2\pi}{N_{0i}}, 0) \times g + (0, \frac{4\pi}{N_{0i}}, 0) \times h = (\frac{\pi}{N_i}g, \frac{2\pi}{N_{0i}}g + \frac{4\pi}{N_{0i}}h, 0) \tag{146}$$

$$(\Theta_{j,i}, \Theta_{0j,i}, \Theta_{00j,i}) = \begin{cases} (\frac{\pi}{N_j}, \frac{2\pi}{N_{0j}}, 0) \times \frac{2N_j}{N_i}l + (0, \frac{4\pi}{N_{0j}}, 0) \times m = (\frac{2\pi}{N_i}l, \frac{4\pi N_j}{N_0 N_i}l + \frac{4\pi}{N_0}m, 0), & \text{if } N_0 \le N_j \text{ and } N_0/2 \le N_i \\ (\frac{\pi}{N_j}, \frac{2\pi}{N_{0j}}, 0) \times \frac{2N_j}{N_i}l + (0, \frac{4\pi}{N_{0j}}, 0) \times \frac{N_0}{2N_i}m = (\frac{2\pi}{N_i}l, \frac{4\pi N_j}{N_0 N_i}l + \frac{2\pi}{N_i}m, 0), & \text{if } N_0 \le N_j \text{ and } N_0/2 > N_i \\ (\frac{\pi}{N_j}, \frac{2\pi}{N_{0j}}, 0) \times \frac{2N_j}{N_i}l + (0, \frac{4\pi}{N_{0j}}, 0) \times m = (\frac{2\pi}{N_i}l, \frac{4\pi}{N_i}l + \frac{2\pi}{N_i}m, 0), & \text{if } N_j \le N_0 \text{ and } N_j/2 = N_i \\ (\frac{\pi}{N_j}, \frac{2\pi}{N_{0j}}, 0) \times \frac{2N_j}{N_i}l + (0, \frac{4\pi}{N_{0j}}, 0) \times \frac{N_j}{2N_i}m = (\frac{2\pi}{N_i}l, \frac{4\pi}{N_i}l + \frac{2\pi}{N_i}m, 0), & \text{if } N_j \le N_0 \text{ and } N_j/2 > N_i \end{cases} \tag{147}$$

By the constraint

$$\begin{cases} \Theta_{ij,0}+\Theta_{oj,i}+\frac{N_j}{N^{oi}}\Theta_{0i,j}=0,\ \frac{2\pi}{N_{0i}}a+\frac{4\pi N_j}{N_0N_i}l+\frac{4\pi}{N_0}m+\frac{N_j}{N^{0i}}(\frac{2\pi}{N_{0i}}g+\frac{4\pi}{N_{0i}}h)=0, & \text{if } N_0\le N_j \text{ and } N_0/2\le N_i \\ \Theta_{ij,0}+\Theta_{oj,i}+\frac{N_j}{N^{oi}}\Theta_{0i,j}=0,\ \frac{2\pi}{N_{0i}}a+\frac{4\pi N_j}{N_0N_i}l+\frac{2\pi}{N_i}m+\frac{N_j}{N^{0i}}(\frac{2\pi}{N_{0i}}g+\frac{4\pi}{N_{0i}}h)=0, & \text{if } N_0\le N_j \text{ and } N_0/2> N_i \\ \frac{N_0}{N_j}\Theta_{ij,0}+\Theta_{oj,i}+\Theta_{0i,j}=0,\ \frac{N_0}{N_j}(\frac{2\pi}{N_i}a)+\frac{4\pi}{N_i}l+\frac{2\pi}{N_i}m+\frac{2\pi}{N_i}g+\frac{4\pi}{N_i}h=0, & \text{if } N_j\le N_0 \text{ and } N_j/2\ge N_i \end{cases} \tag{148}$$

where g, l are chosen as generating phases, only a, m, h need to be considered here.

By the constraint $\Theta_{ij,i}=-\frac{N_j}{2}\Theta_{i,j}$, $\frac{2\pi}{N_i}c=-\frac{N_j}{2}(\frac{\pi}{N_i}g)$, written further as:

$$\begin{cases} \frac{2\pi}{N_i}c=\pi g,\ c=0,\frac{N_i}{2} \pmod{N_i} \text{ depending on } g \pmod{2N_i}, & \text{if } N_j=2N_i \\ \frac{2\pi}{N_i}c=-\frac{N_j}{2}(\frac{\pi}{N_i}g),\ c=0 \pmod{N_i} \text{ and } g \pmod{2N_i}, & \text{if } N_j>2N_i \end{cases} \tag{149}$$

By the constraint $\Theta_{ij,j}=-\Theta_{j,i}$, $\frac{2\pi}{N_i}e=-\frac{2\pi}{N_i}l$, $e=-l \pmod{N_i}$.

Combine all the constraints, the generating phases are:

$$g \pmod{2N_i},\ l \pmod{N_i},\ h \pmod{N_{0i}/2},\ m \pmod{\min\{N_0/2, N_i\}} \tag{150}$$

Hence the classification is $\mathbb{Z}_{2N_i}\times\mathbb{Z}_{N_i}\times\mathbb{Z}_{\min\{N_0/2,N_i\}}\times\mathbb{Z}_{N_{0i}/2}$ (a $\mathbb{Z}_2$ complex fermion layer absorbed into a $\mathbb{Z}_{N_i}\times\mathbb{Z}_{N_i}\times\mathbb{Z}_{\min\{N_0/2,N_i\}}\times\mathbb{Z}_{N_{0i}/2}$ BSPT layer), where the complex fermion layer indicators are:

$$\Theta_{fi,j}=\frac{N_{0i}}{2}\Theta_{0i,j}=\pi g,\ \Theta_{fj,i}=\frac{N_{0j}}{2}\Theta_{0j,i}=0 \tag{151}$$

D. Category (D)

The newly involved constraints in 3D are:

$$N_k\Theta_{ij,k}=0,\quad N_i\Theta_{jk,i}=0,\quad N_j\Theta_{ki,j}=0 \tag{152}$$

$$N_k\Theta_{0ij,k}=0,\quad N_i\Theta_{0jk,i}=0,\quad N_j\Theta_{0ki,j}=0 \tag{153}$$

$$\frac{N^{ijk}}{N^{ij}}\Theta_{ij,k}+\frac{N^{ijk}}{N^{jk}}\Theta_{jk,i}+\frac{N^{ijk}}{N^{ki}}\Theta_{ki,j}=0 \tag{154}$$

$$\Theta_{0ij,k}=\Theta_{ijk,0}=\Theta_{0jk,i}=-\Theta_{0ki,j} \tag{155}$$

$$\Theta_{ijk,i}=\Theta_{iij,k}=\Theta_{ijj,k}=\Theta_{jjk,i}=\Theta_{jkk,i}=\Theta_{kki,j} \tag{156}$$

where subset (D4) can be totally absorbed into (D1), (D2) and (D3).

1. *m is odd*

Set $m=1$ (i.e. $G_f=\mathbb{Z}_2^f\times\mathbb{Z}_{N_i}\times\mathbb{Z}_{N_j}\times\mathbb{Z}_{N_k}$) and assume $N_i\le N_j\le N_k$ without loss of generality.

(1) If $\frac{N_i}{2}, \frac{N_j}{2}, \frac{N_k}{2}$ are all odd (i.e. $\mathbb{Z}_2^f\times\mathbb{Z}_2\times\mathbb{Z}_2\times\mathbb{Z}_2$), invoking the known 2D results and combining with the 3D constraints $N_\sigma\Theta_{\mu,\sigma}=0$, $N_\sigma\Theta_{\mu\nu,\sigma}=0$, $N_\sigma\Theta_{\mu\nu\lambda,\sigma}=0$, the generating phases for the sets (D1), (D2) and (D3) are:

$$(\Theta_{ij,k},\Theta_{0ij,k})=(\frac{\pi}{N_{ij}},\frac{2\pi}{N_{0ij}})\times 2a+(0,\frac{4\pi}{N_{0ij}})=(\pi a,0) \tag{157}$$

$$(\Theta_{jk,i},\Theta_{0jk,i})=(\frac{\pi}{N_{jk}},\frac{2\pi}{N_{0jk}})\times 2b+(0,\frac{4\pi}{N_{0jk}})=(\pi b,0) \tag{158}$$

$$(\Theta_{ki,j},\Theta_{0ki,j})=(\frac{\pi}{N_{ki}},\frac{2\pi}{N_{0ki}})\times 2c+(0,\frac{4\pi}{N_{0ki}})=(\pi c,0) \tag{159}$$

where a, b, c are integers.

By the constraint $\Theta_{ij,k}+\Theta_{jk,i}+\Theta_{ki,j}=0$, $\pi a+\pi b+\pi c=0$.

Hence in this case the classification is $\mathbb{Z}_2\times\mathbb{Z}_2$, which belongs to BSPT.

(2) If $\frac{N_i}{2}, \frac{N_j}{2}$ are odd and $\frac{N_k}{2}$ is even (i.e. $\mathbb{Z}_2^f\times\mathbb{Z}_2\times\mathbb{Z}_2\times\mathbb{Z}_{N_k}$), the generating phases for the sets (D1), (D2) and (D3) are:

$$(\Theta_{ij,k},\Theta_{0ij,k})=(\frac{\pi}{N_{ij}},\frac{2\pi}{N_{0ij}})\times a+(0,\frac{4\pi}{N_{0ij}})=(\frac{\pi}{2}a,\pi a) \tag{160}$$

$$(\Theta_{jk,i},\Theta_{0jk,i})=(\frac{2\pi}{N_{jk}},0)\times b+(0,\frac{2\pi}{N_{0jk}})\times c=(\pi b,\pi c) \tag{161}$$

$$(\Theta_{ki,j},\Theta_{0ki,j})=(\frac{2\pi}{N_{ki}},0)\times d+(0,\frac{2\pi}{N_{0ki}})\times e=(\pi d,\pi e) \tag{162}$$

By the constraint $\frac{N_k}{2}\Theta_{ij,k}+\Theta_{jk,i}+\Theta_{ki,j}=0$, $\frac{N_k}{2}\frac{\pi}{2}a+\pi b+\pi d=0$.

By the constraint $\Theta_{0ij,k}=\Theta_{0jk,i}=-\Theta_{0ki,j}$, $a=c=e \pmod 4$.

Combine the two constraints: $a=c=e \pmod 4$, $b=d$ or $b=d+1 \pmod 2$.

Hence in this case the classification is $\mathbb{Z}_4 \times \mathbb{Z}_2$, which is a $\mathbb{Z}_2$ non-Abelian complex fermion layer absorbed into a $\mathbb{Z}_2 \times \mathbb{Z}_2$ BSPT layer.

(3) If $\frac{N_i}{2}$ is odd and $\frac{N_j}{2}$, $\frac{N_k}{2}$ are even, or $\frac{N_i}{2}$, $\frac{N_j}{2}$, $\frac{N_k}{2}$ are all even (i.e. $\mathbb{Z}_2^f \times \mathbb{Z}_2 \times \mathbb{Z}_{N_j} \times \mathbb{Z}_{N_k}$ or $\mathbb{Z}_2^f \times \mathbb{Z}_{N_i} \times \mathbb{Z}_{N_j} \times \mathbb{Z}_{N_k}$), the generating phases for the sets (D1), (D2) and (D3) are:

$$(\Theta_{ij,k}, \Theta_{0ij,k}) = (\frac{2\pi}{N_{ij}}, 0) \times a + (0, \frac{2\pi}{N_{0ij}}) \times b = (\frac{2\pi}{N_i} a, \pi b) \tag{163}$$

$$(\Theta_{jk,i}, \Theta_{0jk,i}) = (\frac{2\pi}{N_{jk}}, 0) \times \frac{N_j}{N_i} c + (0, \frac{2\pi}{N_{0jk}}) \times d = (\frac{2\pi}{N_i} c, \pi d) \tag{164}$$

$$(\Theta_{ki,j}, \Theta_{0ki,j}) = (\frac{2\pi}{N_{ki}}, 0) \times e + (0, \frac{2\pi}{N_{0ki}}) \times f = (\frac{2\pi}{N_i} e, \pi f) \tag{165}$$

By the constraint $\frac{N_k}{N_j}\Theta_{ij,k} + \Theta_{jk,i} + \Theta_{ki,j} = 0$, $\frac{N_k}{N_j}\frac{2\pi}{N_i} a + \frac{2\pi}{N_i} c + \frac{2\pi}{N_i} e = 0$.

By the constraint $\Theta_{0ij,k} = \Theta_{0jk,i} = -\Theta_{0ki,j}$, $b = d = f$ (mod 2).

Combine the two constraints, the remaining three generating phases are: a (mod N_i), c (mod N_i), $b = d = f$ (mod 2).

Hence in this case the classification is $\mathbb{Z}_{N_i} \times \mathbb{Z}_{N_i} \times \mathbb{Z}_2$ (or $\mathbb{Z}_{N_{ijk}} \times \mathbb{Z}_{N_{ijk}} \times \mathbb{Z}_2$), which is a simple stacking of a $\mathbb{Z}_{N_i} \times \mathbb{Z}_{N_i}$ BSPT layer and a $\mathbb{Z}_2$ non-Abelian complex fermion layer.

2. *m is even*

For symmetry group $\mathbb{Z}_{N_0}^f \times \mathbb{Z}_{N_i} \times \mathbb{Z}_{N_j} \times \mathbb{Z}_{N_k}$, the generating phases for the sets (D1), (D2) and (D3) are:

$$(\Theta_{ij,k}, \Theta_{0ij,k}) = (\frac{2\pi}{N_{ij}}, 0) \times a + (0, \frac{2\pi}{N_{0ij}}) \times \frac{N_{0ij}}{N_{0ijk}} b = (\frac{2\pi}{N_i} a, \frac{2\pi}{N_{0ijk}} b) \tag{166}$$

$$(\Theta_{jk,i}, \Theta_{0jk,i}) = (\frac{2\pi}{N_{jk}}, 0) \times \frac{N_j}{N_i} c + (0, \frac{2\pi}{N_{0jk}}) \times \frac{N_{0jk}}{N_{0ijk}} d = (\frac{2\pi}{N_i} c, \frac{2\pi}{N_{0ijk}} d) \tag{167}$$

$$(\Theta_{ki,j}, \Theta_{0ki,j}) = (\frac{2\pi}{N_{ki}}, 0) \times e + (0, \frac{2\pi}{N_{0ki}}) \times \frac{N_{0ki}}{N_{0ijk}} f = (\frac{2\pi}{N_i} e, \frac{2\pi}{N_{0ijk}} f) \tag{168}$$

By the constraint $\frac{N_k}{N_j}\Theta_{ij,k} + \Theta_{jk,i} + \Theta_{ki,j} = 0$, $\frac{N_k}{N_j}\frac{2\pi}{N_i} a + \frac{2\pi}{N_i} c + \frac{2\pi}{N_i} e = 0$.

By the constraint $\Theta_{0ij,k} = \Theta_{0jk,i} = -\Theta_{0ki,j}$, $b = d = -f$ (mod N_{0ijk}).

Combine the two constraints, the remaining three generating phases are: a (mod N_i), c (mod N_i), $b = d = f$ (mod N_{0ijk}).

Hence in this case the classification is $\mathbb{Z}_{N_i} \times \mathbb{Z}_{N_i} \times \mathbb{Z}_{N_{0ijk}}$, which is a $\mathbb{Z}_2$ complex fermion layer absorbed into a $\mathbb{Z}_{N_i} \times \mathbb{Z}_{N_i} \times \mathbb{Z}_{N_{0ijk}/2}$ BSPT layer if the non-Abelian complex fermion layer indicator is $\Theta_{fij,k} = m\Theta_{0ij,k} = \pi b$ (mod 2), while it is simply a $\mathbb{Z}_{N_i} \times \mathbb{Z}_{N_i} \times \mathbb{Z}_{N_{0ijk}}$ BSPT layer if the non-Abelian complex fermion layer indicator is $\Theta_{fij,k} = m\Theta_{0ij,k} = 0$.

V. SUPPLEMENTARY NOTE 5: STACKING ADDITIVITY OF TOPOLOGICAL INVARIANTS

A. Physical argument

In this section, we give a justification for the additivity of topological invariants under stacking of FSPT states. A mathematical justification on additivity is given in Ref. 7 due to the linearity of all constraints. Here, we give a more detailed microscopic justification based on the following physical picture. If stacking is done after gauging, then it is obvious that the topological invariants, being Abelian Berry phases, must be additive. However, stacking is done before gauging, so additivity cannot be immediately observed. The following discussion applies to both bosonic and fermionic systems.

We recall that the gauging procedure of Ref. 14 (also see Appendix A of Ref. 1 for a general description) has a special property: the gauge flux operator on *every* plaquette of the lattice commutes with the gauged Hamiltonian. That is, flux on every plaquette is conserved. Accordingly, eigenstates of the gauged system are all of the form $|\Psi_{\mathrm{SPT}}(\phi)\rangle \otimes |\Psi_{\mathrm{gauge}}(\phi)\rangle$, where ϕ is a fixed flux configuration, $|\Psi_{\mathrm{SPT}}(\phi)\rangle$ describes the state of the SPT degrees of freedom in the presence of ϕ, and $|\Psi_{\mathrm{gauge}}(\phi)\rangle$ describes the state of the gauge field.

For a given eigenstate, we now adiabatically deform the state in a cyclic fashion and assume that an Abelian Berry phase Θ results, which may be any of the topological invariants defined in this paper. There are two contributions, Θ_{SPT} and Θ_{gauge}, from $|\Psi_{\mathrm{SPT}}(\phi)\rangle$ and $|\Psi_{\mathrm{gauge}}(\phi)\rangle$ respectively. The total Berry phase is $\Theta = \Theta_{\mathrm{SPT}} + \Theta_{\mathrm{gauge}}$. The gauging procedure in Ref. 14 is designed in a way such that $\Theta_{\mathrm{gauge}} = 0$. In general, Θ_{gauge} does not have to be zero, e.g., if the gauge field has a Chern-Simon type interaction. However, for our purpose, the gauge field is a tool to detect SPT physics, so it is a natural choice not to introduce any topological Berry phase that is purely due to the gauge field. In fact, $\Theta_{\mathrm{gauge}} = 0$ is implicitly assumed in almost all works that use the method of gauging symmetries in the field of symmetry-protected and symmetry-enriched topological phases.

With the above understanding, we now consider stacking two SPT systems a and b. For individual systems, the Berry phases associated with an adiabatic process is

$\Theta^a = \Theta^a_{\text{SPT}}$ and $\Theta^b = \Theta^b_{\text{SPT}}$, respectively. After stacking, eigenstates of the gauged system are of the form $|\Psi^a_{\text{SPT}}(\phi)\rangle\otimes|\Psi^b_{\text{SPT}}(\phi)\rangle\otimes|\Psi_{\text{gauge}}(\phi)\rangle$. Therefore, the total Berry phase associated with the same adiabatic process is given by $\Theta^{a+b} = \Theta^a_{\text{SPT}} + \Theta^b_{\text{SPT}} + \Theta_{\text{gauge}} = \Theta^a + \Theta^b$, where the fact $\Theta_{\text{gauge}} = 0$ is used. This is the stacking additivity of topological invariants.

B. Example

As explained above, the stacking additivity of the topological invariants can be verified by the additivity of solutions to the constraints of topological invariants. To better illustrate this additivity, we show the additive structure by "adding" two FSPT phases in the example $G^f = \mathbb{Z}_2^f \times \mathbb{Z}_2 \times \mathbb{Z}_8$. Let the first FSPT phase be associated with the following topological invariants:

$$(\Theta_{0ij,0}, \Theta_{ij,i}, \Theta_{0ij,i}, \Theta_{0ij,j}, \Theta_{i,j}, \Theta_{0i,j}, \Theta_{00i,j}, \Theta_{00j,i}) = (0,0,0,0,\frac{\pi}{2},\pi,0,0), \tag{169}$$

$$(\Theta_{ij,j}, \Theta_{j,i}) = (0,0). \tag{170}$$

One can check the it is a solution to the constraints of topological invariants. Let the second FSPT phase be associated with the following topological invariants:

$$(\Theta_{0ij,0}, \Theta_{ij,i}, \Theta_{0ij,i}, \Theta_{0ij,j}, \Theta_{i,j}, \Theta_{0i,j}, \Theta_{00i,j}, \Theta_{00j,i}) = (\pi,\pi,\pi,\pi,\frac{3\pi}{4},\frac{\pi}{2},\pi,\pi), \tag{171}$$

$$(\Theta_{ij,j}, \Theta_{j,i}) = (0,0). \tag{172}$$

It is again a solution. Stacking these two FSPT phases, we obtain the total topological invariants to be:

$$(\Theta_{0ij,0}, \Theta_{ij,i}, \Theta_{0ij,i}, \Theta_{0ij,j}, \Theta_{i,j}, \Theta_{0i,j}, \Theta_{00i,j}, \Theta_{00j,i}) = (\pi,\pi,\pi,\pi,\frac{5\pi}{4},\frac{3\pi}{2},\pi,\pi), \tag{173}$$

$$(\Theta_{ij,j}, \Theta_{j,i}) = (0,0). \tag{174}$$

One can check again that it satisfies all of the 14 constraints. Therefore, the added new phase is again a valid FSPT phase with the symmetry group $G^f = \mathbb{Z}_2^f\times\mathbb{Z}_2\times\mathbb{Z}_8$.

VI. SUPPLEMENTARY NOTE 6: CLASSIFICATION OF 3D FSPT PHASES WITH UNITARY FINITE ABELIAN G_f USING GENERAL GROUP SUPER-COHOMOLOGY THEORY

In this section, we will derive the classification of 3D FSPT with unitary finite Abelian G_f, using the general group super-cohomology theory[9–11]. We first give a short review of general group super-cohomology theory of FSPT phases in section VI A. Some useful group cohomology calculations and relations for cocycles are given in section VI B. After that, the detailed calculations are given in sections VI C and VI D for non-extended and extended unitary finite Abelian G_f FSPT, respectively.

A. Review of general group super-cohomology theory

The general group super-cohomology theory for FSPT phases is developed in[9–11]. The classification data for 3D FSPT with *unitary* symmetry group $G_f = \mathbb{Z}_2^f \times_{\omega_2} G_b$ is a triple of cochains (n_2, n_3, ν_4). The data $n_2 \in H^2(G_b,\mathbb{Z}_2)/\Gamma^2$ specifies the Majorana chain decorations to the intersection lines of G_b-domain walls. The n_3 data is a cochain in $C^3(G_b,\mathbb{Z}_2)/B^3(G_b,\mathbb{Z}_2)/\Gamma^3$, specifying the complex fermion decorations to the intersection points of G_b-domain walls. And the last $\nu_4 \in C^4(G_b,U(1))/B^3(G_b,U(1))/\Gamma^4$ is the usual bosonic SPT classification data. These classification data satisfy the twisted cocycle equations:

$$\mathrm{d}n_2 = 0, \tag{175}$$
$$\mathrm{d}n_3 = \omega_2 \smile n_2 + n_2 \smile n_2, \tag{176}$$
$$\mathrm{d}\nu_4 = \mathcal{O}_5[n_3], \tag{177}$$

where the most general expression of the last obstruction function is

$$\begin{aligned}\mathcal{O}_5[n_3](012345) = &(-1)^{(\omega_2\smile n_3+n_3\smile_1 n_3+n_3\smile_2 \mathrm{d}n_3)(012345)+\omega_2(013)\mathrm{d}n_3(12345)} \\ &\times(-1)^{\mathrm{d}n_3(02345)\mathrm{d}n_3(01235)+\omega_2(023)[\mathrm{d}n_3(01245)+\mathrm{d}n_3(01235)+\mathrm{d}n_3(01234)]} \\ &\times i^{\mathrm{d}n_3(01245)\mathrm{d}n_3(01234)\ (\mathrm{mod}\ 2)}\times(-i)^{[\mathrm{d}n_3(12345)+\mathrm{d}n_3(02345)+\mathrm{d}n_3(01345)]\mathrm{d}n_3(01235)\ (\mathrm{mod}\ 2)}.\end{aligned} \tag{178}$$

All the classification data is defined modulo trivialization subgroup Γ^i. For state labelled by these data, we can construct a symmetric gapped state without topological order on their boundary. Therefore, they are in fact trivial FSPT states[12]. For unitary Abelian G_f, the trivialization groups Γ^i can be calculated from

$$\Gamma^2 = \{\omega_2 \smile n_0 \in H^2(G_b,\mathbb{Z}_2) | n_0 \in H^0(G_b,\mathbb{Z})\}, \tag{179}$$

$$\Gamma^3 = \{\omega_2 \smile n_1 + (\omega_2 \smile_1 \omega_2)\lfloor n_0/2 \rfloor \in H^3(G_b,\mathbb{Z}_2) | n_1 \in H^1(G_b,\mathbb{Z}_2), n_0 \in H^0(G_b,\mathbb{Z}_T)\}, \tag{180}$$

$$\Gamma^4 = \{(-1)^{\omega_2\smile n_2 + n_2\smile n_2} \in H^4(G_b,U(1)) | n_2 \in H^2(G_b,\mathbb{Z}_2)\}. \tag{181}$$

In the rest of this section, we will use the above equations to derive a complete classification for unitary finite Abelian G_f FSPT phases.

B. Cohomology groups, explicit cocycles and Bockstein homomorphism

There are many useful relations for cocycles of the cyclic group $\mathbb{Z}_N$. They can tremendously simplify the FSPT calculations. In the following, we denote the cyclic group as $\mathbb{Z}_N = \{0,1,...,N-1\}$, where the group multiplication is given by the addition of integers mod N. We will use the notation $\overset{n}{=}$ to mean equality up to mod n. Similarly, $\overset{\mathbb{Z}}{=}$ emphasizes an equality in the ring of integers. And $\overset{n,\mathrm{d}}{=}$ means an equality up to $\mathbb{Z}_n$-valued coboundaries (a.k.a. they belong to the same $\mathbb{Z}_n$-valued cohomology class). The symbol $\lfloor x \rfloor$ is the floor function as the largest integer smaller than or equal to x. And $[x]_N$ is defined to be the mod N value of an integer x.

1. $\mathbb{Z}_2$-coefficient cohomology

The cohomology ring for $\mathbb{Z}_N$ (N even) with $\mathbb{Z}_2$ coefficient is

$$H^*(\mathbb{Z}_N,\mathbb{Z}_2) = \begin{cases} \mathbb{Z}_2[n_1^{\mathbb{Z}_2}], & \text{if } N \overset{4}{=} 2, \\ \mathbb{Z}_2[n_1^{\mathbb{Z}_2}, n_2^{\mathbb{Z}_2}]/\{(n_1^{\mathbb{Z}_2})^2\}, & \text{if } N \overset{4}{=} 0. \end{cases} \tag{182}$$

So we can use the cup product of $n_1^{\mathbb{Z}_2} \in H^1(\mathbb{Z}_N,\mathbb{Z}_2)$ and $n_2^{\mathbb{Z}_2} \in H^2(\mathbb{Z}_N,\mathbb{Z}_2)$ to obtain all cocycles. The superscript $\mathbb{Z}_2$ of $n_i^{\mathbb{Z}_2}$ emphasizes that they are $\mathbb{Z}_2$-valued cocycles. As will shown later, the cocycle $(n_1^{\mathbb{Z}_2})^2 := n_1^{\mathbb{Z}_2} \smile n_1^{\mathbb{Z}_2}$ has different result for different N: $(n_1^{\mathbb{Z}_2})^2 \overset{2,\mathrm{d}}{=} n_2^{\mathbb{Z}_2}$ if $N \overset{4}{=} 2$, and $(n_1^{\mathbb{Z}_2})^2 \overset{2,\mathrm{d}}{=} 0$ if $N \overset{4}{=} 0$.

The explicit cocycles in $H^*(\mathbb{Z}_N,\mathbb{Z}_2)$ are also very useful in the calculations. The expressions of $n_1^{\mathbb{Z}_2}$ and $n_2^{\mathbb{Z}_2}$ are ($a,b \in \mathbb{Z}_N$):

$$n_1^{\mathbb{Z}_2}(a) \overset{2}{=} [a]_2, \tag{183}$$

$$n_2^{\mathbb{Z}_2}(a,b) \overset{2}{=} \left\lfloor \frac{a+b}{N} \right\rfloor \overset{2}{=} \frac{a+b-[a+b]_N}{N}. \tag{184}$$

Other cocycles can be obtained from the cup products of $n_1^{\mathbb{Z}_2}$ and $n_2^{\mathbb{Z}_2}$.

2. $\mathbb{Z}$-coefficient cohomology

The $\mathbb{Z}$-coefficient group cohomology for $\mathbb{Z}_N$ is

$$H^*(\mathbb{Z}_N,\mathbb{Z}) = \mathbb{Z}[n_2^{\mathbb{Z}}]/\{N n_2^{\mathbb{Z}}\} = \mathbb{Z}_N[n_2^{\mathbb{Z}}]. \tag{185}$$

Again, the superscript $\mathbb{Z}$ of the generator 2-cocycle $n_2^{\mathbb{Z}}$ is to emphasize that it is $\mathbb{Z}$-valued. In fact, the $\mathbb{Z}_2$-valued cocycle $n_2^{\mathbb{Z}_2}$ in Eq. (184) is the same as $\mathbb{Z}$-valued cocycle $n_2^{\mathbb{Z}}$:

$$n_2^{\mathbb{Z}}(a,b) \overset{\mathbb{Z}}{=} \left\lfloor \frac{a+b}{N} \right\rfloor \overset{\mathbb{Z}}{=} \frac{a+b-[a+b]_N}{N} \overset{\mathbb{Z}}{=} \left(\frac{1}{N}\mathrm{d}n_1^{\mathbb{Z}_N}\right)(a,b), \tag{186}$$

where

$$n_1^{\mathbb{Z}_N}(a) \overset{N}{=} [a]_N \tag{187}$$

is the generator of $H^1(\mathbb{Z}_N, \mathbb{Z}_N) = \mathbb{Z}_N$. So it is easy to see that

$$\mathrm{d}n_2^{\mathbb{Z}} \overset{\mathbb{Z}}{=} \mathrm{d}\left(\frac{1}{N}\mathrm{d}n_1^{\mathbb{Z}_N}\right) \overset{\mathbb{Z}}{=} \frac{1}{N}\mathrm{d}^2 n_1^{\mathbb{Z}_N} \overset{\mathbb{Z}}{=} 0, \tag{188}$$

from the fact $\mathrm{d}^2 = 0$. All other cocycles in $H^*(\mathbb{Z}_N, \mathbb{Z})$ can be obtained from the addition and cup product of several $n_2^{\mathbb{Z}}$.

Using the relations of $\mathbb{Z}_2$ and $\mathbb{Z}$-valued cocycles, we can show that

$$(n_1^{\mathbb{Z}_2})^2 := n_1^{\mathbb{Z}_2} \smile n_1^{\mathbb{Z}_2} \overset{\mathbb{Z}}{=} \frac{1}{2}\mathrm{d}n_1^{\mathbb{Z}_2} \overset{\mathbb{Z}}{=} \frac{1}{2}\mathrm{d}n_1^{\mathbb{Z}_N} + \mathrm{d}\mu_1 \overset{\mathbb{Z}}{=} \frac{N}{2}\frac{\mathrm{d}n_1^{\mathbb{Z}_N}}{N} + \mathrm{d}\mu_1 \overset{\mathbb{Z}}{=} \frac{N}{2}n_2^{\mathbb{Z}} + \mathrm{d}\mu_1 \overset{\mathbb{Z},\mathrm{d}}{=} \frac{N}{2}n_2^{\mathbb{Z}}. \tag{189}$$

where we have defined a $\mathbb{Z}$-valued 1-cochain ($a \in \mathbb{Z}_N$):

$$\mu_1(a) := \frac{1}{2}(n_1^{\mathbb{Z}_2} - n_1^{\mathbb{Z}_N})(a) \overset{\mathbb{Z}}{=} \frac{[a]_2 - [a]_N}{2}. \tag{190}$$

Since N is even, the right-hand-side of the above equation is indeed an integer. So we have

$$(n_1^{\mathbb{Z}_2})^2 \overset{\mathbb{Z},\mathrm{d}}{=} \frac{N}{2}n_2^{\mathbb{Z}} \overset{2,\mathrm{d}}{=} \begin{cases} 0, & \text{if } N \overset{4}{=} 0, \\ n_2^{\mathbb{Z}_2}, & \text{if } N \overset{4}{=} 2. \end{cases} \tag{191}$$

This is exactly the result claimed below Eq. (182). When $N \overset{4}{=} 0$, we also know the explicit coboundary as

$$(n_1^{\mathbb{Z}_2})^2 \overset{\mathbb{Z}}{=} \frac{N}{2}n_2^{\mathbb{Z}} + \mathrm{d}\mu_1 \overset{2}{=} \mathrm{d}\mu_1. \tag{192}$$

3. *Bockstein homomorphism*

The notion of Bockstein homomorphism is very useful in checking whether a cocycle $(-1)^{f_k}$ $[f_k \in H^k(G_b, \mathbb{Z}_2)]$ is a $U(1)$-valued coboundary or not. It is defined as a mapping from $H^k(G_b, \mathbb{Z}_2)$ to $H^{k+1}(G_b, \mathbb{Z})$:

$$\beta(f_k) \overset{\mathbb{Z}}{=} \frac{\mathrm{d}f_k}{2}, \tag{193}$$

where f_k is a cocycle in $H^k(G_b, \mathbb{Z}_2)$. The coboundary operator is defined with appropriate plus and minus signs in integers. Because of $\mathrm{d}f_k \overset{2}{=} 0$, the right-hand-side of Eq. (193) is always an integer. The Bockstein homomorphism is the connecting isomorphism between $H^k(G_b, \mathbb{Z}_2)$ and $H^{k+1}(G_b, \mathbb{Z})$. So we have the useful relation

$$(-1)^{f_k} \in B^k(G_b, U(1)) \Longleftrightarrow \beta(f_k) \in B^{k+1}(G_b, \mathbb{Z}). \tag{194}$$

We can use it to check whether $(-1)^{f_k}$ is a U(1)-valued coboundary or not.

When acting on the cup product of two cocycles, the Bockstein homomorphism reads

$$\beta(x \smile y) \overset{\mathbb{Z}}{=} \beta(x) \smile y + (-1)^{\deg(x)} x \smile \beta(y), \tag{195}$$

which is essentially the Leibniz's rule for coboundary operators. When modulo two, the Bockstein homomorphism is related to Steenrod square operation and higher cup product as

$$\beta(f_k) \overset{2}{=} Sq^1(f_k) \overset{2}{=} f_k \smile_{k-1} f_k. \tag{196}$$

There are also some useful equations for Steenrod squares:

$$Sq^0(x) \overset{2,\mathrm{d}}{=} x, \tag{197}$$

$$Sq^i(x) \overset{2,\mathrm{d}}{=} 0, \quad \text{if } i > \deg(x), \tag{198}$$

$$Sq^i(x) \overset{2,\mathrm{d}}{=} x \smile x, \quad \text{if } i = \deg(x), \tag{199}$$

$$Sq^n(x \smile y) \overset{2,\mathrm{d}}{=} \sum_{i+j=n} Sq^i(x) \smile Sq^j(y). \tag{200}$$

The last equation is called Cartan formula.

For the special case of cohomology for group $\mathbb{Z}_N$, the Bockstein homomorphism of the generator $n_2^{\mathbb{Z}_2} \in H^2(\mathbb{Z}_N, \mathbb{Z}_2) = \mathbb{Z}_2$ can be shown to be zero:

$$Sq^1(n_2^{\mathbb{Z}_2}) \overset{2}{=} \beta(n_2^{\mathbb{Z}_2}) \overset{\mathbb{Z}}{=} \frac{1}{2}\mathrm{d}n_2^{\mathbb{Z}_2} \overset{\mathbb{Z}}{=} \frac{1}{2}\mathrm{d}^2 n_1^{\mathbb{Z}} \overset{\mathbb{Z}}{=} 0, \tag{201}$$

where we used $\mathrm{d}^2 = 0$ in the last step.

C. Classification of FSPT with $G_f = \mathbb{Z}_2^f \times \prod_{i=1}^K \mathbb{Z}_{N_i}$

The fermionic symmetry group $G_f = \mathbb{Z}_2^f \times \prod_{i=1}^K \mathbb{Z}_{N_i}$ is associated with bosonic symmetry group

$$G_b = \prod_{i=1}^K \mathbb{Z}_{N_i}, \tag{202}$$

and trivial central extension $\omega_2 = 0$. It is known that, for a given positive integer N, we have a unique factorization $N = \prod_p p^{n_p}$ (p is a prime number and n_p is a positive integer in $\mathbb{Z}_+$) and a group isomorphism $\mathbb{Z}_N \cong \prod_p \mathbb{Z}_{p^{n_p}}$. For prime number $p > 2$, the cohomology group $H^*(\mathbb{Z}_{p^{n_p}}, \mathbb{Z}_2)$ is trivial. Therefore, the symmetry group $\prod_{p>2} \mathbb{Z}_{p^{n_p}}$ can only protect bosonic SPT phases, and can only affect the FSPT classifications though adding some BSPT phases. To understand genuine FSPT, we can assume

$$N_i = 2^{k_i} \quad (1 \le i \le K,\ k_i \in \mathbb{Z}_+) \tag{203}$$

in the bosonic symmetry group G_b Eq. (202). Without loss of generality, we can also reorder the Abelian groups such that

$$N_i \le N_{i+1} \quad (1 \le i \le K-1). \tag{204}$$

Using Künneth formula and universal coefficient theorem, the relevant cohomology groups with $\mathbb{Z}_2$ and $U(1)$ coefficients for Eq. (202) ($N_i = 2^{k_i} \ge 2$, $N_i \le N_{i+1}$) are given by

$$H^2(G_b, \mathbb{Z}_2) = \mathbb{Z}_2^{C_K^1 + C_K^2} = \prod_{1\le i\le K} \langle n_2^{(i)} \rangle \prod_{1\le i<j\le K} \langle n_1^{(i)} n_1^{(j)} \rangle, \tag{205}$$

$$H^3(G_b, \mathbb{Z}_2) = \mathbb{Z}_2^{C_K^1 + 2C_K^2 + C_K^3} = \prod_{1\le i\le K} \langle n_1^{(i)} n_2^{(i)} \rangle \prod_{1\le i<j\le K} \langle n_1^{(i)} n_2^{(j)}, n_2^{(i)} n_1^{(j)} \rangle \prod_{1\le i<j<k\le K} \langle n_1^{(i)} n_1^{(j)} n_1^{(k)} \rangle, \tag{206}$$

$$H^4(G_b, U(1)) = \prod_{1\le i<j\le K} \mathbb{Z}_{N_{ij}}^2 \prod_{1\le i<j<k\le K} \mathbb{Z}_{N_{ijk}}^2 \prod_{1\le i<j<k<l\le K} \mathbb{Z}_{N_{ijkl}}, \tag{207}$$

$$\begin{aligned} H^5(G_b, U(1)) = H^6(G_b, \mathbb{Z}) &= \prod_{1\le i\le K} \mathbb{Z}_{N_i} \prod_{1\le i<j\le K} \mathbb{Z}_{N_{ij}}^2 \prod_{1\le i<j<k\le K} \mathbb{Z}_{N_{ijk}}^4 \prod_{1\le i<j<k<l\le K} \mathbb{Z}_{N_{ijkl}}^3 \prod_{1\le i<j<k<l<m\le K} \mathbb{Z}_{N_{ijklm}} \\ &= \prod_{1\le i\le K} \langle (n_2^{(i)})^3 \rangle \prod_{1\le i<j\le K} \langle (n_2^{(i)})^2 n_2^{(j)}, n_2^{(i)} (n_2^{(j)})^2 \rangle \prod_{1\le i<j<k\le K} \langle n_2^{(i)} n_2^{(j)} n_2^{(k)} \rangle \times ... \end{aligned} \tag{208}$$

Here, $C_K^i = \frac{K!(K-i)!}{i!}$ is the binomial coefficient. We have listed the generators for the $\mathbb{Z}_2$ coefficient cohomology groups, as well as the generators of some some relevant subgroups of $\mathbb{Z}$ coefficient cohomology groups. They are expressed as cup products of $n_1^{(i)}$ and $n_2^{(i)}$ ($1 \le i \le K$), which are generating cocycles for the i-th Abelian group $\mathbb{Z}_{N_i}$ in G_b. In the following, all $n_p^{(i)}$ are $\mathbb{Z}_2$-valued p-cocycles with the superscript $\mathbb{Z}_2$ omitted.

1. *Trivialization*

Since $\omega_2 = 0$, we have the trivialization groups $\Gamma^2 = 0$ and $\Gamma^3 = 0$ according to Eqs. (179) and (180). So all nontrivial obstruction-free n_2 and n_3 correspond to nontrivial FSPT states.

The trivialization group Γ^4 is generated $(-1)^{n_2\smile n_2}=(-1)^{Sq^2(n_2)}$ according to Eq. (181). We have two choices of root 2-cocycle $n_2\in H^2(G_b,\mathbb{Z}_2)$ [see Eq. (205)]: $n_2=n_2^{(i)}$ and $n_2=n_1^{(i)}n_1^{(j)}$ for some $1\le i<j\le K$. For both of them, one can show that $\beta(Sq^2(n_2))\overset{\mathbb{Z},\mathrm{d}}{=}0$:

$$\beta(Sq^2(n_2^{(i)}))\overset{\mathbb{Z},\mathrm{d}}{=}\beta(n_2^{(i)}n_2^{(i)})\overset{\mathbb{Z},\mathrm{d}}{=}\beta(n_2^{(i)})n_2^{(i)}+n_2^{(i)}\beta(n_2^{(i)})\overset{\mathbb{Z}}{=}0, \tag{209}$$

$$\beta(Sq^2(n_1^{(i)}n_1^{(j)}))\overset{\mathbb{Z},\mathrm{d}}{=}\beta(Sq^1(n_1^{(i)})Sq^1(n_1^{(j)}))\overset{\mathbb{Z},\mathrm{d}}{=}\beta((n_1^{(i)})^2(n_1^{(j)})^2)\overset{\mathbb{Z},\mathrm{d}}{=}0, \tag{210}$$

where we used Eq. (201) and $\beta((n_1^{(i)})^2)\overset{\mathbb{Z},\mathrm{d}}{=}\beta(n_1^{(i)})n_1^{(i)}-n_1^{(i)}\beta(n_1^{(i)})\overset{\mathbb{Z},\mathrm{d}}{=}(n_1^{(i)})^3-(n_1^{(i)})^3\overset{\mathbb{Z}}{=}0$. Therefore, we have $(-1)^{n_2\smile n_2}\overset{\mathrm{d}}{=}1$ for all $n_2\in H^2(G_b,\mathbb{Z}_2)$. The trivialization group Γ^4 is also trivial.

2. *Obstruction*

To solve the equations Eqs. (176) and (177), we have to check that the right-hand-side of the equations are coboundaries, otherwise there are no solutions. We need to check these obstructions layer by layer: we first solve Eq. (176) for n_3 with a given 2-cocycle n_2; after obtaining n_3, we can solve Eq. (177) for ν_4 with this n_3.

(1) Obstruction for n_2.

The equation for n_3 is Eq. (176), i.e., $\mathrm{d}n_3\overset{2}{=}n_2\smile n_2$ (recall that $\omega_2=0$). So the obstruction function for n_2 is

$$\mathcal{O}_4[n_2]\overset{2}{=}n_2\smile n_2. \tag{211}$$

Below we will check the obstructions for all possible $n_2\in H^2(G_b,\mathbb{Z}_2)$. In fact, we only need to check the obstructions for generators of n_2. All others can be obtained from the cohomology operation property: $\mathcal{O}_4[n_2+n_2']\overset{2,\mathrm{d}}{=}\mathcal{O}_4[n_2]+\mathcal{O}_4[n_2']$.

(1.1) $n_2\overset{2}{=}n_2^{(i)}$ ($1\le i\le K$) [obstructed].

According to Eq. (182), $\mathcal{O}_4[n_2^{(i)}]=n_2^{(i)}\smile n_2^{(i)}$ is always a nontrivial 4-cocycle in $H^4(G_b,\mathbb{Z}_2)$. So $n_2=n_2^{(i)}$ is obstructed for all $1\le i\le K$.

(1.2) $n_2\overset{2}{=}n_1^{(i)}n_1^{(j)}$ ($1\le i<j\le K$) [obstruction-free iff $N_j\ge 4$].

For $n_2\overset{2}{=}n_1^{(i)}n_1^{(j)}$ ($1\le i<j\le K$), one can show that

$$\mathcal{O}_4[n_2]\overset{2}{=}n_1^{(i)}n_1^{(j)}n_1^{(i)}n_1^{(j)} \tag{212}$$

$$\overset{2}{=}n_1^{(i)}\left[n_1^{(i)}n_1^{(j)}+\mathrm{d}\left(n_1^{(i)}\smile_1 n_1^{(j)}\right)\right]n_1^{(j)} \tag{213}$$

$$\overset{2}{=}(n_1^{(i)})^2(n_1^{(j)})^2+\mathrm{d}\left[n_1^{(i)}\left(n_1^{(i)}\smile_1 n_1^{(j)}\right)n_1^{(j)}\right], \tag{214}$$

where we have used Steenrod's higher cup product $\smile_i$[13]. It can be used to switch the cup product of two cocycles as

$$\mathrm{d}(n_p\smile_i n_q)\overset{2}{=}\mathrm{d}n_p\smile_i n_q+n_p\smile_i \mathrm{d}n_q+n_p\smile_{i-1}n_q+n_q\smile_{i-1}n_p. \tag{215}$$

From Eq. (191), we can further simplify $\mathcal{O}_4[n_2]$ as $\mathcal{O}_4[n_2]\overset{2,\mathrm{d}}{=}n_2^{(i)}n_2^{(j)}$ if $N_i=N_j=2$, and $\mathcal{O}_4[n_2]\overset{2,\mathrm{d}}{=}0$, if $N_j\ge 4$ (note that we have assumed $N_i\le N_j$). For the latter case ($N_j\ge 4$), we also have

$$\mathcal{O}_4[n_2]\overset{2}{=}(n_1^{(i)})^2(n_1^{(j)})^2+\mathrm{d}\left[n_1^{(i)}\left(n_1^{(i)}\smile_1 n_1^{(j)}\right)n_1^{(j)}\right] \tag{216}$$

$$\overset{2}{=}(n_1^{(i)})^2\mathrm{d}\mu_1^{(j)}+\mathrm{d}\left[n_1^{(i)}\left(n_1^{(i)}\smile_1 n_1^{(j)}\right)n_1^{(j)}\right] \tag{217}$$

$$\overset{2}{=}\mathrm{d}\left[(n_1^{(i)})^2\mu_1^{(j)}+n_1^{(i)}\left(n_1^{(i)}\smile_1 n_1^{(j)}\right)n_1^{(j)}\right] \tag{218}$$

where $\mu_1^{(j)}$ is the cochain defined in Eq. (190) for the subgroup $\mathbb{Z}_{N_j}$. So we can get a special solution of n_3 as

$$\bar{n}_3\overset{2}{=}(n_1^{(i)})^2\mu_1^{(j)}+n_1^{(i)}\left(n_1^{(i)}\smile_1 n_1^{(j)}\right)n_1^{(j)}. \tag{219}$$

In summary, $n_2\overset{2}{=}n_1^{(i)}n_1^{(j)}$ ($1\le i<j\le K$) is obstructed by $\mathcal{O}_4[n_2]$ iff $N_i=N_j=2$. For other cases (i.e., $N_j\ge 4$), $\mathrm{d}n_3\overset{2}{=}n_2\smile n_2$ has a special n_3 solution Eq. (219).

(2) Obstruction for n_3.

After checked the obstruction function $\mathcal{O}_4[n_2]$, we now can check the obstruction function $\mathcal{O}_5[n_3]$. There are two cases we need to calculate. The first case (2.0) below is that $n_2 = n_1^{(i)} n_1^{(j)}$ for some $1 \le i < j \le K$ and $N_j \ge 4$, and n_3 has a special solution $\bar{n}_3$ shown in Eq. (219). We then need to calculate the full complicated obstruction function $\mathcal{O}_5[\bar{n}_3]$ in Eq. (178). The second case (from 2.1 to 2.4) is associated with $n_2 \overset{2}{=} 0$, and a 3-cocycle n_3 in $H^3(G_b, \mathbb{Z}_2)$. And the obstruction function in this case is merely

$$\mathcal{O}_5[n_3]\Big|_{\omega_2 \overset{2}{=} 0, n_2 \overset{2}{=} 0} = (-1)^{Sq^2(n_3)} = (-1)^{n_3 \smile_1 n_3}, \tag{220}$$

for $\mathrm{d}n_3 \overset{2}{=} n_2 \smile n_2 \overset{2}{=} 0$.

(2.0) $n_2 \overset{2}{=} n_1^{(i)} n_1^{(j)}$ $(1 \le i < j \le K,\ N_j \ge 4)$ and $n_3 \overset{2}{=} \bar{n}_3$ [obstruction-free iff $N_i N_j \ge 16$].

In this case, we should use the obstruction function $\mathcal{O}_5[\bar{n}_3]$ in Eq. (178) which involves some non-higher-cup-product terms. Therefore, we can use the complete U(1) cocyle invariants for unitary finite Abelian groups to check whether $\mathcal{O}_5[\bar{n}_3]$ is trivial or not. After some tedious calculations, the possibly nontrivial invariants associated with $\mathcal{O}_5[\bar{n}_3]$ are

$$e^{i\Omega_{ij}} = i^{-N^{ij}(N^{ij}-1)N_j(N_j-1)/4}, \tag{221}$$

$$e^{i\Omega_{ji}} = i^{N^{ij}(N^{ij}-1)N_i(N_i-1)/4}. \tag{222}$$

For all the invariants to be trivial and $\bar{n}_3$ to be obstruction-free, we need $N^{ij} N_i/4 \overset{4}{=} 0$. Recall that $N_i \le N_j$, so the obstruction-free condition reduces to

$$N_i N_j \ge 16. \tag{223}$$

In summary, the classification data $(n_2 \overset{2}{=} n_1^{(i)} n_1^{(j)}, n_3 \overset{2}{=} \bar{n}_3)$ is obstruction-free $(1 \le i < j \le K)$, iff the parameters of the symmetry group satisfy $N_i N_j \ge 16$. For $G_b = \mathbb{Z}_2 \times \mathbb{Z}_2$, it is obstructed by $\mathcal{O}_4[n_2]$. For $G_b = \mathbb{Z}_2 \times \mathbb{Z}_4$, $\mathcal{O}_5[n_3]$ is nontrivial although $\mathcal{O}_4[n_2]$ is trivial.

For the following cases, we have $n_2 \overset{2}{=} 0$. So we can use the simpler obstruction function Eq. (220).

(2.1) $n_3 \overset{2}{=} n_1^{(i)} n_2^{(i)}$ $(1 \le i \le K)$ [obstructed].

Using Eq. (201) and the formulas Eqs. (197) and (200) related to Steenrod square, we have $Sq^2(n_3) \overset{2}{=} Sq^2(n_1^{(i)} n_2^{(i)}) \overset{2}{=} n_1^{(i)} (n_2^{(i)})^2$. We can use the Bockstein homomorphism of $Sq^2(n_3)$ is

$$\beta[Sq^2(n_3)] \overset{\mathbb{Z}}{=} \beta[n_1^{(i)} (n_2^{(i)})^2] \overset{\mathbb{Z}}{=} (n_1^{(i)})^2 (n_2^{(i)})^2 \overset{\mathbb{Z},\mathrm{d}}{=} \frac{N_i}{2} (n_2^{(i)})^3, \tag{224}$$

which is the $\frac{N_i}{2}$-th nontrivial cocycle in $H^6(\mathbb{Z}_{N_i}, \mathbb{Z}) = \mathbb{Z}_{N_i} = \langle (n_2^{(i)})^3 \rangle$. So $n_3 \overset{2}{=} n_1^{(i)} n_2^{(i)}$ is obstructed.

(2.2) $n_3 \overset{2}{=} n_1^{(i)} n_2^{(j)}$ $(1 \le i < j \le K)$ [obstructed].

Similar to the previous case, we have $Sq^2(n_3) \overset{2}{=} Sq^2(n_1^{(i)} n_2^{(j)}) \overset{2}{=} n_1^{(i)} (n_2^{(j)})^2$. The Bockstein homomorphism is then

$$\beta[Sq^2(n_3)] \overset{\mathbb{Z}}{=} \beta[n_1^{(i)} (n_2^{(j)})^2] \overset{\mathbb{Z}}{=} (n_1^{(i)})^2 (n_2^{(j)})^2 \overset{\mathbb{Z},\mathrm{d}}{=} \frac{N_i}{2} n_2^{(i)} (n_2^{(j)})^2. \tag{225}$$

The generator $n_2^{(i)} (n_2^{(j)})^2$ of $\mathbb{Z}_{N_{ij}} \subset H^6(\mathbb{Z}_{N_i} \times \mathbb{Z}_{N_j}, \mathbb{Z})$ [see Eq. (208)] has order $N_{ij} = N_i$ (note that $N_i \le N_j$). Therefore, $\beta[Sq^2(n_3)]$ is a nontrivial cocycle in $H^6(\mathbb{Z}_{N_i} \times \mathbb{Z}_{N_j}, \mathbb{Z})$, and $n_3 \overset{2}{=} n_1^{(i)} n_2^{(j)}$ is obstructed.

(2.3) $n_3 \overset{2}{=} n_2^{(i)} n_1^{(j)}$ $(1 \le i < j \le K)$ [obstruction-free iff $N_i < N_j$].

In this case, we have $Sq^2(n_3) \overset{2}{=} Sq^2(n_2^{(i)} n_1^{(j)}) \overset{2}{=} (n_2^{(i)})^2 n_1^{(j)}$. Its Bockstein homomorphism is

$$\beta[Sq^2(n_3)] \overset{\mathbb{Z}}{=} \beta[(n_2^{(i)})^2 n_1^{(j)}] \overset{\mathbb{Z}}{=} (n_2^{(i)})^2 (n_1^{(j)})^2 \overset{\mathbb{Z},\mathrm{d}}{=} \frac{N_j}{2} (n_2^{(i)})^2 n_2^{(j)}. \tag{226}$$

The generator $(n_2^{(i)})^2 n_2^{(j)}$ of $\mathbb{Z}_{N_{ij}} \subset H^6(\mathbb{Z}_{N_i} \times \mathbb{Z}_{N_j}, \mathbb{Z})$ [see Eq. (208)] also has order $N_{ij} = N_i$. If $N_i = N_j$, Eq. (226) is a nontrivial cocycle. If $N_i < N_j$, then Eq. (226) is a coboundary, since the coefficient $N_j/2$ is a multiple of the order N_i.

In summary, $n_3 \overset{2}{=} n_2^{(i)} n_1^{(j)}$ is obstruction-free iff $N_i < N_j$. We can also derive the explicit ν_4.

(2.4) $n_3 \overset{2}{=} n_1^{(i)} n_1^{(j)} n_1^{(k)}$ $(1 \le i < j < k \le K)$ [obstruction-free iff $N_k \ge 4$].

In this case, we can show that

$$Sq^2(n_3) \stackrel{2}{=} Sq^2[n_1^{(i)}n_1^{(j)}n_1^{(k)}] \stackrel{2}{=} (n_1^{(i)})^2(n_1^{(j)})^2n_1^{(k)} + (n_1^{(i)})^2n_1^{(j)}(n_1^{(k)})^2 + n_1^{(i)}(n_1^{(j)})^2(n_1^{(k)})^2, \tag{227}$$

$$\beta[Sq^2(n_3)] \stackrel{\mathbb{Z}}{=} 3(n_1^{(i)})^2(n_1^{(j)})^2(n_1^{(k)})^2 \stackrel{\mathbb{Z},\mathrm{d}}{=} \frac{3N_iN_jN_k}{8}n_2^{(i)}n_2^{(j)}n_2^{(k)}. \tag{228}$$

The generator $n_2^{(i)}n_2^{(j)}n_2^{(k)}$ in the subgroup $\mathbb{Z}_{N_{ijk}} \subset H^6(G_b,\mathbb{Z})$ has order N_{ijk} [see Eq. (208)]. So $n_3 \stackrel{2}{=} n_1^{(i)}n_1^{(j)}n_1^{(k)}$ is obstruction-free iff $\frac{3N_iN_jN_k}{8} \in \mathbb{Z}$. Using the fact $2 \le N_i \le N_j \le N_k$, the obstruction-free condition is reduced to $N_k \ge 4$.

3. Summary

Note that all the obstruction functions are different for the above generators n_3 that are obstructed. Therefore, the summation of several generators, as a generic 3-cocycle $n_3 \in H^3(G_b,\mathbb{Z}_2)$, is obstructed if one of the generator is obstructed. To summarize, the obstruction-free classification data (n_2,n_3,ν_4) belongs to the groups:

$$n_2 \in \prod_{1\le i<j\le K} \begin{cases} \mathbb{Z}_2, & (N_iN_j \ge 16) \\ 0, & (N_iN_j \le 8) \end{cases}, \tag{229}$$

$$n_3 \in \prod_{1\le i<j\le K} \begin{cases} \mathbb{Z}_2, & (N_i < N_j) \\ 0, & (N_i = N_j) \end{cases} \times \prod_{1\le i<j<k\le K} \begin{cases} \mathbb{Z}_2, & (N_k \ge 4) \\ 0, & (N_i = N_j = N_k = 2) \end{cases}, \tag{230}$$

$$\nu_4 \in \prod_{1\le i<j\le K} \mathbb{Z}_{N_{ij}}^2 \prod_{1\le i<j<k\le K} \mathbb{Z}_{N_{ijk}}^2 \prod_{1\le i<j<k<l\le K} \mathbb{Z}_{N_{ijkl}}. \tag{231}$$

D. Classification of FSPT with $G_f = \mathbb{Z}_{2m}^f \times \prod_{i=1}^K \mathbb{Z}_{N_i}$

With the definition

$$N_0 = 2m, \tag{232}$$

the fermionic symmetry group $G_f = \mathbb{Z}_{2m}^f \times \prod_{i=1}^K \mathbb{Z}_{N_i}$ can be also written as $G_f = \prod_{i=0}^K \mathbb{Z}_{N_i}$. It is associated with bosonic symmetry group

$$G_b = \mathbb{Z}_m \times \prod_{i=1}^K \mathbb{Z}_{N_i} = \mathbb{Z}_{N_0/2} \times \prod_{i=1}^K \mathbb{Z}_{N_i}. \tag{233}$$

and extension specified by

$$\omega_2(a,b) \stackrel{2}{=} n_2^{(0)}(a,b) \stackrel{2}{=} \left\lfloor \frac{a_0+b_0}{m} \right\rfloor. \tag{234}$$

Without loss of generality, we can assume $m = 2^k$ to be the k-th power of 2 ($k \ge 1$). Otherwise $\mathbb{Z}_{2m}^f$ is isomorphic to $\mathbb{Z}_{2^{k+1}}^f \times \mathbb{Z}_{m/2^k}$, and the latter subgroup can be absorbed to $\mathbb{Z}_{N_i}$ with $i > 0$. We note that $n_2^{(0)}$ is the nontrivial $\mathbb{Z}_2$-valued 2-cocycle of $\mathbb{Z}_m$, rather than that of $\mathbb{Z}_{N_0} = \mathbb{Z}_{2m}$. This is different from $n_2^{(i)}$ for $1 \le i \le K$.

Similar to the previous section, we assume

$$N_0 = 2^{k_0} \quad (k_0 \ge 2), \tag{235}$$

$$N_i = 2^{k_i} \quad (0 \le i \le K,\ k_i \ge 1). \tag{236}$$

Without loss of generality, we can also reorder the Abelian groups such that

$$N_i \le N_{i+1} \quad (1 \le i \le K-1). \tag{237}$$

Using Künneth formula and universal coefficient theorem, the relevant cohomology groups with $\mathbb{Z}_2$ and $U(1)$ coefficients for Eq. (233) are given by

$$H^2(G_b,\mathbb{Z}_2)=\mathbb{Z}_2^{1+2C_K^1+C_K^2}=\langle n_2^{(0)}\rangle\prod_{1\le i\le K}\langle n_2^{(i)},n_1^{(0)}n_1^{(i)}\rangle\prod_{1\le i<j\le K}\langle n_1^{(i)}n_1^{(j)}\rangle,\tag{238}$$

$$\begin{aligned}H^3(G_b,\mathbb{Z}_2)=\mathbb{Z}_2^{1+3C_K^1+3C_K^2+C_K^3}&=\langle n_1^{(0)}n_2^{(0)}\rangle\prod_{1\le i\le K}\langle n_1^{(i)}n_2^{(i)},n_1^{(0)}n_2^{(i)},n_1^{(i)}n_2^{(0)}\rangle\\&\times\prod_{1\le i<j\le K}\langle n_1^{(i)}n_2^{(j)},n_2^{(i)}n_1^{(j)},n_1^{(0)}n_1^{(i)}n_1^{(j)}\rangle\prod_{1\le i<j<k\le K}\langle n_1^{(i)}n_1^{(j)}n_1^{(k)}\rangle,\end{aligned}\tag{239}$$

$$\begin{aligned}H^4(G_b,U(1))=&\prod_{1\le i\le K}\mathbb{Z}^2_{\gcd(m,N_i)}\prod_{1\le i<j\le K}\mathbb{Z}^2_{N_{ij}}\mathbb{Z}^2_{\gcd(m,N_{ij})}\prod_{1\le i<j<k\le K}\mathbb{Z}^2_{N_{ijk}}\mathbb{Z}_{\gcd(m,N_{ijk})}\prod_{1\le i<j<k<l\le K}\mathbb{Z}_{N_{ijkl}},\\
=&\prod_{1\le i\le K}\langle e^{2\pi i\frac{k_{0i}}{\gcd(m,N_i)}n_1^{(0)}n_2^{(0)}n_1^{(i)}},e^{2\pi i\frac{k_{i0}}{\gcd(m,N_i)}n_1^{(i)}n_2^{(i)}n_1^{(0)}}\rangle\prod_{1\le i<j\le K}\langle e^{2\pi i\frac{k_{ij}}{N_{ij}}n_1^{(i)}n_2^{(i)}n_1^{(j)}},e^{2\pi i\frac{k_{ji}}{N_{ij}}n_1^{(j)}n_2^{(j)}n_1^{(i)}}\rangle\\
&\times\prod_{1\le i<j\le K}\langle e^{2\pi i\frac{k_{0ij}}{\gcd(m,N_i)}n_1^{(0)}n_1^{(i)}n_2^{(j)}},e^{2\pi i\frac{k_{0ji}}{\gcd(m,N_j)}n_1^{(0)}n_1^{(j)}n_2^{(i)}}\rangle\prod_{1\le i<j<k\le K}\langle e^{2\pi i\frac{k_{ijk}}{N_{ij}}n_1^{(i)}n_1^{(j)}n_2^{(k)}},e^{2\pi i\frac{k_{ikj}}{N_{ik}}n_1^{(i)}n_1^{(k)}n_2^{(j)}}\rangle\\
&\times\prod_{1\le i<j<k\le K}\langle e^{2\pi i\frac{k_{0ijk}}{\gcd(m,N_{ijk})}n_1^{(0)}n_1^{(i)}n_1^{(j)}n_1^{(k)}}\rangle\prod_{1\le i<j<k<l\le K}\langle e^{2\pi i\frac{k_{ijkl}}{N_{ijkl}}n_1^{(i)}n_1^{(j)}n_1^{(k)}n_1^{(l)}}\rangle.\end{aligned}\tag{240}$$

We have also listed explicitly the "cononical" U(1)-valued cocycles, in terms of $n_1^{(i)}\in H^1(\mathbb{Z}_{N_i},\mathbb{Z}_{N_i})$ and $n_2^{(i)}\in H^2(\mathbb{Z}_{N_i},\mathbb{Z})$ [see Eqs. (187) and (186) for their expressions]. The parameters k's are integers modulo the corresponding subgroup orders:

$$k_{0i},k_{i0}\in\gcd(m,N_i),\qquad k_{ij},k_{ji}\in N_{ij},\qquad k_{0ij},k_{0ji}\in\gcd(m,N_{ij}),\tag{241}$$

$$k_{ijk},k_{ikj}\in N_{ijk},\qquad k_{0ijk}\in\gcd(m,N_{ijk}),\qquad k_{ijkl}\in\gcd(m,N_{ijk}).\tag{242}$$

There cohomology results are essentially the same as the previous Eq. (205) to Eq. (207). The only difference is that we have one more subgroup $\mathbb{Z}_{N_0/2}=\mathbb{Z}_m$ in G_b. The cohomology group of $H^5(G_b,U(1))$ can be similarly obtained.

1. *Trivialization*

Since $\omega_2=n_2^{(0)}$, we have the trivialization group

$$\Gamma^2=\mathbb{Z}_2=\langle n_2^{(0)}\rangle,\tag{243}$$

according to Eq. (179). As $n_2^{(0)}\smile_1 n_2^{(0)}\overset{2,\mathrm{d}}{=}Sq^1(n_2^{(0)})\overset{2,\mathrm{d}}{=}0$, we have trivialization group

$$\Gamma^3=\mathbb{Z}_2^{K+1}=\prod_{0\le i\le K}\langle n_2^{(0)}n_1^{(i)}\rangle,\tag{244}$$

according to Eq. (180).

The trivialization group Γ^4 in Eq. (181) is much more complicated. We have several choices of n_2 in Eq. (238). (1) For $n_2\overset{2}{=}n_2^{(i)}$ $(0\le i\le K)$, the trivialization cocycle $(-1)^{\omega_2n_2+n_2n_2}=(-1)^{n_2^{(0)}n_2^{(i)}+n_2^{(i)}n_2^{(i)}}$ is a U(1) coboundary. This is because $\beta(n_2^{(0)}n_2^{(i)}+n_2^{(i)}n_2^{(i)})\overset{\mathbb{Z}}{=}0$, as $\beta(n_2^{(i)})\overset{\mathbb{Z}}{=}0$ from Eq. (201). (2) For $n_2\overset{2}{=}n_1^{(0)}n_1^{(i)}$ $(1\le i\le K)$, we have

$$(-1)^{\omega_2n_2+n_2n_2}=(-1)^{n_2^{(0)}n_1^{(0)}n_1^{(i)}+n_1^{(0)}n_1^{(i)}n_1^{(0)}n_1^{(i)}}\overset{\mathrm{d}}{=}(-1)^{n_2^{(0)}n_1^{(0)}n_1^{(i)}+(n_1^{(0)})^2(n_1^{(i)})^2}\tag{245}$$

$$\overset{\mathrm{d}}{=}(-1)^{n_2^{(0)}n_1^{(0)}n_1^{(i)}}=e^{2\pi i\frac{\gcd(m,N_i)/2}{\gcd(m,N_i)}n_2^{(0)}n_1^{(0)}n_1^{(i)}}\tag{246}$$

which is a nontrivial element in $H^5(\mathbb{Z}_m\times\mathbb{Z}_{N_i},U(1))$ compared to the explicit cocycles in Eq. (240). (3) For $n_2\overset{2}{=}n_1^{(i)}n_1^{(j)}$, we can do similar calculations and find that

$$(-1)^{\omega_2n_2+n_2n_2}\overset{\mathrm{d}}{=}(-1)^{n_2^{(0)}n_1^{(i)}n_1^{(j)}}=e^{2\pi i\frac{N_{ij}/2}{N_{ij}}n_2^{(0)}n_1^{(i)}n_1^{(j)}}.\tag{247}$$

This cocycle is a coboundary in $\mathbb{Z}_{\gcd(m,N_{ij})} \subset H^4(\mathbb{Z}_m\times\mathbb{Z}_{N_i}\times\mathbb{Z}_{N_j}, U(1))$, iff $N_{ij}/2 \in \gcd(m, N_{ij})\mathbb{Z}$, which is equivalent to $m < N_{ij}$. In summary, the trivialization group for BSPT 4-cocycles is

$$\Gamma^4 = \mathbb{Z}_2^K \times \prod_{1\le i<j\le K} \begin{cases} \mathbb{Z}_2, & (m \ge N_{ij}) \\ \mathbb{Z}_1, & (m < N_{ij}) \end{cases} = \prod_{1\le i\le K} \langle (-1)^{n_2^{(0)} n_1^{(0)} n_1^{(i)}} \rangle \prod_{1\le i<j\le K \text{ and } m\ge N_{ij}} \langle (-1)^{n_2^{(0)} n_1^{(i)} n_1^{(j)}} \rangle. \tag{248}$$

We note that all the nontrivial elements in the trivialization group are of order two.

2. *Obstruction*

Now we check the obstructions for different choices of n_2 and n_3 shown in Eqs. (238) and (239).

(1) Obstruction for n_2.

From the equation of n_3 Eq. (176), the obstruction function for n_2 is

$$\mathcal{O}_4[n_2] \stackrel{2}{=} \omega_2 \smile n_2 + n_2 \smile n_2. \tag{249}$$

Again, we need to check the obstructions for generators of n_2. All others can be obtained from $\mathcal{O}_4[n_2 + n_2'] \stackrel{2,\mathrm{d}}{=} \mathcal{O}_4[n_2] + \mathcal{O}_4[n_2']$.

(1.1) $n_2 \stackrel{2}{=} n_2^{(0)}$ [trivialized].

Although $n_2 \stackrel{2}{=} n_2^{(0)}$ is obstruction-free, it is trivialized by Γ^2 in Eq. (243).

(1.2) $n_2 \stackrel{2}{=} n_2^{(i)}$ $(1 \le i \le K)$ [obstructed].

The obstruction function $\mathcal{O}_4[n_2^{(i)}] \stackrel{2}{=} n_2^{(0)} \smile n_2^{(i)} + n_2^{(i)} \smile n_2^{(i)}$ is a nontrivial 4-cocycle in $H^4(G_b, \mathbb{Z}_2)$. So $n_2 = n_2^{(i)}$ is obstructed for all $1 \le i \le K$.

(1.3) $n_2 \stackrel{2}{=} n_1^{(0)} n_1^{(i)}$ $(1 \le i \le K)$ [obstructed].

In this case, the obstruction function is

$$\mathcal{O}_4[n_2] \stackrel{2}{=} n_2^{(0)} n_1^{(0)} n_1^{(i)} + n_1^{(0)} n_1^{(i)} n_1^{(0)} n_1^{(i)} \stackrel{2,\mathrm{d}}{=} n_2^{(0)} n_1^{(0)} n_1^{(i)} + (n_1^{(0)})^2 (n_1^{(i)})^2. \tag{250}$$

The first part $n_2^{(0)} n_1^{(0)} n_1^{(i)}$ is always a nontrivial cocycle and can not be cancelled by the second. So $n_2 \stackrel{2}{=} n_1^{(0)} n_1^{(i)}$ are all obstructed for $(1 \le i \le K)$.

(1.4) $n_2 \stackrel{2}{=} n_1^{(i)} n_1^{(j)}$ $(1 \le i < j \le K)$ [obstructed].

Similar to the previous case, the obstruction function is

$$\mathcal{O}_4[n_2] \stackrel{2}{=} n_2^{(0)} n_1^{(i)} n_1^{(j)} + n_1^{(i)} n_1^{(j)} n_1^{(i)} n_1^{(j)} \stackrel{2,\mathrm{d}}{=} n_2^{(0)} n_1^{(i)} n_1^{(j)} + (n_1^{(i)})^2 (n_1^{(j)})^2. \tag{251}$$

The first part is always nontrivial, and can not be cancelled by the second. So $n_2 \stackrel{2}{=} n_1^{(i)} n_1^{(j)}$ are all obstructed for $(1 \le i < j \le K)$.

In summary, all nontrivial n_2 (even for the summation of some generators of n_2 discussed above) are either trivialized or obstructed. So there is no Majorana chain decoration for FSPT with arbitrary unitary finite Abelian symmetry group G_f, if the symmetry is extended by $\omega_2 \ne 0$. The only possibility is complex fermion decoration layer n_3 which will be discussed below.

(2) Obstruction for n_3.

Since all nontrivial n_2 are trivialized or obstructed, we only need to consider $n_2 \stackrel{2}{=} 0$. Then the obstruction function $\mathcal{O}_5[n_3]$ in Eq. (178) becomes a simpler form

$$\mathcal{O}_5[n_3]\Big|_{n_2 \stackrel{2}{=} 0} = (-1)^{\omega_2 n_3 + Sq^2(n_3)} = (-1)^{\omega_2 n_3 + n_3 \smile_1 n_3}. \tag{252}$$

(2.1) $n_3 \stackrel{2}{=} n_1^{(0)} n_2^{(0)}$ [trivialized].

The complex fermion decoration data $n_3 \stackrel{2}{=} n_1^{(0)} n_2^{(0)}$ is trivialized by Eq. (244).

(2.2) $n_3 \stackrel{2}{=} n_1^{(i)} n_2^{(i)}$ $(1 \le i \le K)$ [obstructed].

Since $Sq^2(n_3) \stackrel{2}{=} Sq^2(n_1^{(i)} n_2^{(i)}) \stackrel{2}{=} n_1^{(i)} (n_2^{(i)})^2$, we have obstruction function Eq. (252) as

$$\mathcal{O}_5[n_3] = (-1)^{n_2^{(0)} n_1^{(i)} n_2^{(i)} + n_1^{(i)} (n_2^{(i)})^2} = e^{2\pi i \frac{N_i/2}{N_i} n_2^{(0)} n_1^{(i)} n_2^{(i)} + 2\pi i \frac{N_i/2}{N_i} n_1^{(i)} (n_2^{(i)})^2}. \tag{253}$$

The second term $e^{2\pi i \frac{N_i/2}{N_i} n_1^{(i)} (n_2^{(i)})^2}$ is always a nontrivial element in $H^5(\mathbb{Z}_{N_i}, U(1)) = \mathbb{Z}_{N_i} = \langle e^{2\pi i \frac{1}{N_i} n_1^{(i)} (n_2^{(i)})^2} \rangle$. And it cannot be cancelled by the first term. Therefore, $n_3 \overset{2}{=} n_1^{(i)} n_2^{(i)}$ is always obstructed.

(2.3) $n_3 \overset{2}{=} n_1^{(0)} n_2^{(i)}$ $(1 \le i \le K)$ *[obstruction-free iff* $m > N_i$*].*

In this case, we have $Sq^2(n_3) \overset{2}{=} Sq^2(n_1^{(0)} n_2^{(i)}) \overset{2}{=} n_1^{(0)} (n_2^{(i)})^2$, and

$$\mathcal{O}_5[n_3] = (-1)^{n_2^{(0)} n_1^{(0)} n_2^{(i)} + n_1^{(0)} (n_2^{(i)})^2} = e^{2\pi i \frac{m/2}{m} n_2^{(0)} n_1^{(0)} n_2^{(i)} + 2\pi i \frac{m/2}{m} n_1^{(0)} (n_2^{(i)})^2}. \tag{254}$$

The cohomology group of $H^5(\mathbb{Z}_m \times \mathbb{Z}_{N_i})$ has subgroups $\mathbb{Z}_{\gcd(m,N_i)}^2 = \langle e^{2\pi i \frac{1}{m} n_2^{(0)} n_1^{(0)} n_2^{(i)}}, e^{2\pi i \frac{1}{m} n_1^{(0)} (n_2^{(i)})^2} \rangle$. So $\mathcal{O}_5[n_3]$ is a trivial cocycle iff $m/2 \in \gcd(m, N_i)\mathbb{Z}$, which is equivalent to $m > N_i$. Therefore, $n_3 \overset{2}{=} n_1^{(0)} n_2^{(i)}$ $(1 \le i \le K)$ is obstruction-free iff $m > N_i$.

(2.4) $n_3 \overset{2}{=} n_2^{(0)} n_1^{(i)}$ $(1 \le i \le K)$ *[trivialized].*

The complex fermion decoration data $n_3 \overset{2}{=} n_2^{(0)} n_1^{(i)}$ is trivialized by Eq. (244).

(2.5) $n_3 \overset{2}{=} n_1^{(i)} n_2^{(j)}$ $(1 \le i < j \le K)$ *[obstructed].*

We have $Sq^2(n_3) \overset{2}{=} Sq^2(n_1^{(i)} n_2^{(j)}) \overset{2}{=} n_1^{(i)} (n_2^{(j)})^2$, and

$$\mathcal{O}_5[n_3] = (-1)^{n_2^{(0)} n_1^{(i)} n_2^{(j)} + n_1^{(i)} (n_2^{(j)})^2} = e^{2\pi i \frac{N_i/2}{N_i} n_2^{(0)} n_1^{(i)} n_2^{(j)} + 2\pi i \frac{N_i/2}{N_i} n_1^{(i)} (n_2^{(j)})^2}. \tag{255}$$

The cohomology group $H^5(G,U(1))$ has subgroups $\mathbb{Z}_{\gcd(m,N_{ij})} = \langle e^{2\pi i \frac{1}{N_i} n_2^{(0)} n_1^{(i)} n_2^{(j)}} \rangle$ and $\mathbb{Z}_{N_{ij}} = \langle e^{2\pi i \frac{1}{N_i} n_1^{(i)} (n_2^{(j)})^2} \rangle$. So $\mathcal{O}_5[n_3]$ is trivial iff $N_i/2 \in \gcd(m, N_{ij})\mathbb{Z}$ and $N_i/2 \in N_{ij}\mathbb{Z}$. This is impossible since we have assumed $N_i \le N_j$. Therefore, $n_3 \overset{2}{=} n_1^{(i)} n_2^{(j)}$ $(1 \le i < j \le K)$ is always obstructed.

(2.6) $n_3 \overset{2}{=} n_1^{(j)} n_2^{(i)}$ $(1 \le i < j \le K)$ *[obstruction-free iff* $N_i < N_j$*].*

This case is similar to the above one with i and j switched. So the conclusion is that, $\mathcal{O}_5[n_3]$ is trivial iff $N_j/2 \in \gcd(m, N_{ij})\mathbb{Z}$ and $N_j/2 \in N_{ij}\mathbb{Z}$. These conditions are satisfied iff $N_i < N_j$ (note that we have assumed $N_i \le N_j$). Therefore, $n_3 \overset{2}{=} n_1^{(j)} n_2^{(i)}$ $(1 \le i < j \le K)$ is obstruction-free iff $N_i < N_j$.

(2.7) $n_3 \overset{2}{=} n_1^{(0)} n_1^{(i)} n_1^{(j)}$ $(1 \le i < j \le K)$ *[obstructed].*

Using the properties of Steenrod square, we have

$$Sq^2(n_3) \overset{2}{=} Sq^2[n_1^{(0)} n_1^{(i)} n_1^{(j)}] \overset{2}{=} (n_1^{(0)})^2 (n_1^{(i)})^2 n_1^{(j)} + (n_1^{(0)})^2 n_1^{(i)} (n_1^{(j)})^2 + n_1^{(0)} (n_1^{(i)})^2 (n_1^{(j)})^2. \tag{256}$$

The obstruction function is

$$\begin{aligned}
\mathcal{O}_5[n_3] &= (-1)^{n_2^{(0)} n_1^{(0)} n_1^{(i)} n_1^{(j)} + (n_1^{(0)})^2 (n_1^{(i)})^2 n_1^{(j)} + (n_1^{(0)})^2 n_1^{(i)} (n_1^{(j)})^2 + n_1^{(0)} (n_1^{(i)})^2 (n_1^{(j)})^2} && (257)\\
&= (-1)^{n_2^{(0)} n_1^{(0)} n_1^{(i)} n_1^{(j)} + (n_1^{(0)})^2 (n_1^{(i)})^2 n_1^{(j)}} \times e^{(\pi i/2)\mathrm{d}[n_1^{(0)} n_1^{(i)} (n_1^{(j)})^2 + n_1^{(0)} n_1^{(i)} (n_1^{(j)})^2]} && (258)\\
&\overset{\mathrm{d}}{=} (-1)^{n_2^{(0)} n_1^{(0)} n_1^{(i)} n_1^{(j)} + (n_1^{(0)})^2 (n_1^{(i)})^2 n_1^{(j)}} && (259)\\
&= e^{2\pi i \frac{\gcd(m,N_{ij})/2}{\gcd(m,N_{ij})} n_2^{(0)} n_1^{(0)} n_1^{(i)} n_1^{(j)} + \pi i (n_1^{(0)})^2 (n_1^{(i)})^2 n_1^{(j)}}. && (260)
\end{aligned}$$

The first term $e^{2\pi i \frac{\gcd(m,N_{ij})/2}{\gcd(m,N_{ij})} n_2^{(0)} n_1^{(0)} n_1^{(i)} n_1^{(j)}}$ is always a nontrivial cocycle in the subgroup $\mathbb{Z}_{\gcd(m,N_{ij})}$ [of $H^5(G_b, U(1))$] generated by $e^{2\pi i \frac{1}{\gcd(m,N_{ij})} n_2^{(0)} n_1^{(0)} n_1^{(i)} n_1^{(j)}}$. So $n_3 \overset{2}{=} n_1^{(0)} n_1^{(i)} n_1^{(j)}$ $(1 \le i < j \le K)$ is always obstructed.

(2.8) $n_3 \overset{2}{=} n_1^{(i)} n_1^{(j)} n_1^{(k)}$ $(1 \le i < j < k \le K)$ *[obstruction-free iff* $m < N_{ijk}$*].*

Similar to the previous case, we have

$$Sq^2(n_3) \overset{2}{=} Sq^2[n_1^{(i)} n_1^{(j)} n_1^{(k)}] \overset{2}{=} (n_1^{(i)})^2 (n_1^{(j)})^2 n_1^{(k)} + (n_1^{(i)})^2 n_1^{(j)} (n_1^{(k)})^2 + n_1^{(i)} (n_1^{(j)})^2 (n_1^{(k)})^2. \tag{261}$$

The obstruction function is

$$\begin{aligned}
\mathcal{O}_5[n_3] &= (-1)^{n_2^{(0)} n_1^{(i)} n_1^{(j)} n_1^{(k)} + (n_1^{(i)})^2 (n_1^{(j)})^2 n_1^{(k)} + (n_1^{(i)})^2 n_1^{(j)} (n_1^{(k)})^2 + n_1^{(i)} (n_1^{(j)})^2 (n_1^{(k)})^2} && (262)\\
&\overset{\mathrm{d}}{=} (-1)^{n_2^{(0)} n_1^{(i)} n_1^{(j)} n_1^{(k)} + (n_1^{(i)})^2 (n_1^{(j)})^2 n_1^{(k)}} && (263)\\
&= e^{2\pi i \frac{N_{ijk}/2}{N_{ijk}} n_2^{(0)} n_1^{(i)} n_1^{(j)} n_1^{(k)} + 2\pi i \frac{N_i N_j N_k/8}{N_k} n_2^{(i)} n_2^{(j)} n_1^{(k)}}. && (264)
\end{aligned}$$

The cohomology group $H^5(G_b, U(1))$ has subgroups $\mathbb{Z}_{\gcd(m,N_{ijk})}$ generated by $e^{2\pi i \frac{1}{N_{ijk}} n_2^{(0)} n_1^{(i)} n_1^{(j)} n_1^{(k)}}$ and $\mathbb{Z}_{N_{ijk}}$ generated by $e^{2\pi i \frac{1}{N_k} n_2^{(i)} n_2^{(j)} n_1^{(k)}}$. So $\mathcal{O}_5[n_3]$ is a trivial cocycle iff $N_{ijk}/2 \in \gcd(m, N_{ijk})\mathbb{Z}$ and $N_i N_j N_k/8 \in N_{ijk}\mathbb{Z}$. Using the fact $2 \le N_i \le N_j \le N_k$, these conditions are equivalent to $m < N_i$ and $N_j N_k/8 \in \mathbb{Z}$. As $m \ge 2$, they can be further simplified to $m < N_i$ only. In summary, $n_3 \stackrel{2}{=} n_1^{(i)} n_1^{(j)} n_1^{(k)}$ $(1 \le i < j < k \le K)$ is obstruction-free iff $m < N_i$.

3. Summary

We note that all the obstruction functions are different for the above obstructed generating n_3's. So the summation of several generators is obstructed if one of the generator is obstructed. In summary, the trivialization-free and obstruction-free classification data $(n_2 = 0, n_3, \nu_4)$ belongs to the groups:

$$n_3 \in \prod_{1\le i\le K} \begin{cases} \mathbb{Z}_2, & (m > N_i) \\ 0, & (m \le N_i) \end{cases} \times \prod_{1\le i<j\le K} \begin{cases} \mathbb{Z}_2, & (N_i < N_j) \\ 0, & (N_i = N_j) \end{cases} \times \prod_{1\le i<j<k\le K} \begin{cases} \mathbb{Z}_2, & (m < N_{ijk}) \\ 0, & (m \ge N_{ijk}) \end{cases}, \tag{265}$$

$$\begin{aligned} \nu_4 \in & \prod_{1\le i\le K} \mathbb{Z}_{\gcd(m,N_i)} \mathbb{Z}_{\gcd(m,N_i)/2} \times \prod_{1\le i<j\le K} \mathbb{Z}_{N_{ij}}^2 \mathbb{Z}_{\gcd(m,N_{ij})} \times \begin{cases} \mathbb{Z}_{\gcd(m,N_{ij})/2}, & (m \ge N_{ij}) \\ \mathbb{Z}_{\gcd(m,N_{ij})}, & (m < N_{ij}) \end{cases} \\ & \times \prod_{1\le i<j<k\le K} \mathbb{Z}_{N_{ijk}}^2 \mathbb{Z}_{\gcd(m,N_{ijk})} \times \prod_{1\le i<j<k<l\le K} \mathbb{Z}_{N_{ijkl}}. \end{aligned} \tag{266}$$

* These two authors contributed equally to this work.
† cjwang@hku.hk
‡ zcgu@phy.cuhk.edu.hk

1 C. Wang and M. Levin, Phys. Rev. B **91**, 165119 (2015).
2 A. Kitaev, Annals of Physics **321**, 2 (2006).
3 J. Preskill, Caltech Lecture Notes (1999).
4 J. K. Pachos, *Introduction to topological quantum computation* (Cambridge University Press, 2012).
5 P. H. Bonderson, *Non-Abelian anyons and interferometry*, Ph.D. thesis, California Institute of Technology (2007).
6 S. Eliëns, Master's thesis, Universiteit van Amsterdam, Netherlands (2010).
7 C. Wang, C.-H. Lin, and Z.-C. Gu, Phys. Rev. B **95**, 195147 (2017).
8 C. Wang and M. Levin, Phys. Rev. Lett. **113**, 080403 (2014).
9 Z.-C. Gu and X.-G. Wen, Phys. Rev. B **90**, 115141 (2014).
10 Q.-R. Wang and Z.-C. Gu, Phys. Rev. X **8**, 011055 (2018).
11 Q.-R. Wang and Z.-C. Gu, Phys. Rev. X **10**, 031055 (2020).
12 Q.-R. Wang, Y. Qi, and Z.-C. Gu, ArXiv e-prints (2018), arXiv:1810.12899 [cond-mat.str-el].
13 N. E. Steenrod, Annals of Mathematics **48**, 290 (1947).
14 M. Levin and Z.-C. Gu, Phys. Rev. B **86**, 115109 (2012).
